# Qualitative concept elicitation and cognitive debriefing interviews of symptoms, impacts and selected customized PROMIS^®^ Short Forms: a study in patients with axial spondyloarthritis

**DOI:** 10.1186/s41687-023-00575-x

**Published:** 2023-04-20

**Authors:** Amy Findley, Jessica M. Middlehurst, Chloe A. Howse, Molly J. Clifford, William Neill, Sophi Tatlock, Wen-Hung Chen, Marguerite G. Bracher, Dharm S. Patel

**Affiliations:** 1 Adelphi Values Patient-Centered Outcomes, Bollington, Cheshire UK; 2 GSK, Global Value Evidence & Outcomes, Collegeville, PA USA; 3 GSK, Global Value Evidence & Outcomes, Stevenage, Hertfordshire UK; 4 Present Address: 7i Group Limited, Alderley Edge, Cheshire UK

**Keywords:** Qualitative, Concept elicitation, Cognitive debriefing, axSpA

## Abstract

**Background:**

Sleep disturbance, pain, and fatigue are key symptoms/impacts of axial spondyloarthritis (axSpA). Three customized Patient-Reported Outcomes Measurement Information System (PROMIS^®^) Short Forms (Sleep Disturbance, Pain Interference, and Fatigue) have been proposed for use in axSpA to assess these key disease concepts. This study was designed to further understand the patient experience of axSpA and evaluate the content validity of the three customized PROMIS^®^ Short Forms to support their use in axSpA clinical trials.

**Methods:**

Non-interventional, cross-sectional, qualitative (concept elicitation [CE] and cognitive debriefing [CD]) study. Participants took part in 90-min telephone interviews. The CE section used open-ended questions to elicit information about axSpA symptoms and impacts. The CD section involved a ‘think-aloud’ exercise where participants read out each instruction, item, and response option for the customized PROMIS^®^ Short Forms and shared their feedback. Participants also discussed the relevance of the items, response options and recall period. Verbatim interview transcripts were subject to thematic and content analysis.

**Results:**

In total, there were 28 participants (non-radiographic axSpA, n = 12; ankylosing spondylitis, n = 16), from the US (n = 20) and Germany (n = 8). Mean age was 52.8 years, and 57% were male; mean time since diagnosis was 9.5 years. The CE section identified 12 distinct symptoms that characterized axSpA: pain, sleep problems, fatigue/tiredness, stiffness, swelling, vision/eye issues, restricted body movements, headache/migraine, spasms, change in posture/stature, balance/coordination problems, and numbness. Pain, sleep problems, and fatigue/tiredness were experienced by ≥ 90% of participants, occurring simultaneously and exacerbating one another. Participants reported axSpA impacted their lives across six domains of health-related quality of life (HRQoL): physical functioning (100%), emotional wellbeing (89%), work/volunteering (79%), social functioning (75%), activities of daily living (61%) and cognitive functioning (54%). Impacts were most frequently associated with pain, stiffness, and fatigue. CD showed the PROMIS^®^ instruments were conceptually comprehensive and well understood, with all items relevant to ≥ 50% of participants.

**Conclusions:**

Pain, sleep problems and fatigue are pivotal symptoms of axSpA and associated with HRQoL impacts. These results were used to update a conceptual model of axSpA which was originally developed based on a targeted literature review. Interpretability and content validity of the customized PROMIS^®^ Short Forms were confirmed, with each deemed to adequately assess key impacts associated with axSpA, making them suitable for use in axSpA clinical trials.

**Supplementary Information:**

The online version contains supplementary material available at 10.1186/s41687-023-00575-x.

## Plain language summary

Axial spondyloarthritis (axSpA) is a type of arthritis that causes inflammation in the spine and the joints of the lower back. Pain, sleep problems, and fatigue (feeling tired/weak) are common symptoms of axSpA. To measure how well a drug works in clinical trials, patients may be asked to fill in surveys about their symptoms. Surveys called customized PROMIS^®^ Short Forms can be used to measure how healthy patients are. They have been used to assess pain, sleep problems and fatigue in clinical trials for other types of arthritis. They may also be useful for axSpA clinical trials. In this study, we wanted to better understand how patients experience axSpA. We also looked into how easily understood and relevant the questions in the customized PROMIS^®^ Short Forms are for patients with axSpA. We interviewed 28 patients with axSpA by phone. Patients talked about their symptoms of axSpA, and how these affected their lives. At least 90% experienced pain, sleep problems and fatigue. Patients also experienced stiffness, swelling, vision/eye issues, restricted body movements, headache/migraine, spasms, change in posture, balance/coordination problems and numbness. Patients generally found the customized PROMIS^®^ Short Forms were easy to understand and the questions were relevant to their experience of axSpA. This supports using the customized PROMIS^®^ Short Forms in clinical trials for axSpA drugs.

**Supplementary Information:**

The online version contains supplementary material available at 10.1186/s41687-023-00575-x.

## Background

Axial spondyloarthritis (axSpA) is a chronic autoimmune disease characterized by inflammation of the sacroiliac joints and spine [1]. As identified in the Assessment of SpondyloArthritis international Society (ASAS) criteria, patients with axSpA are classified as having either radiographic (r-axSpA, or ankylosing spondylitis [AS]) or non-radiographic (nr-axSpA) disease depending on whether damage to the sacroiliac joints is visible via X-ray [2].

Patients with nr-axSpA and AS report impaired quality of life, and experience a substantial burden of disease [3–5]. Sleep disturbance, pain and fatigue have been consistently reported by patients as key symptoms that greatly impact their performance in daily activities and other functions [6–9]. Prior to this study, we performed a targeted literature review to investigate key symptoms and impacts of axSpA, which highlighted pain interference, sleep disturbance and fatigue as key measurement concepts.

Several patient-reported outcome (PRO) instruments have been developed for use in axSpA, including the Bath Ankylosing Spondylitis Disease Activity Index (BASDAI) [10], the Bath Ankylosing Spondylitis Functional Index (BASFI) [11], the Ankylosing Spondylitis Quality of Life (ASQOL) questionnaire [12], and the Work Productivity and Activity Impairment Questionnaire in AS (WPAI-SpA) [13]. Other, more generic PRO instruments are also frequently used to measure disease impact in axSpA, including the 36-item Short Form Survey (SF-36) and the EuroQol 5 Dimensions (EQ-5D) [14]. These PRO tools provide a broad understanding of the patient’s health status and their disease symptoms, but do not specifically focus on the burden associated with sleep disturbance, pain interference, and fatigue in axSpA. In addition, most of the existing axSpA PRO instruments have been developed specifically for an r-axSpA/AS population, and have limitations such as dichotomous yes/no response options on the ASQoL, which could force respondents to select an answer that does not accurately reflect their experience [12, 15].

Three customized Patient-Reported Outcomes Measurement Information System (PROMIS^®^) Short Forms (Sleep Disturbance, Pain Interference, and Fatigue) were developed for use in rheumatoid arthritis (RA) [16]. As previous qualitative research has shown that the key measurement concepts of pain, sleep and fatigue are similar between patients with RA and axSpA [17–19], these customized PROMIS^®^ Short Forms have been proposed for use in axSpA to assess key disease concepts. At the time of this study, no PRO measures assessing pain interference, sleep disturbance and fatigue have been evaluated in the axSpA patient population via cognitive interviews. This is an important and necessary step to ensure the PRO measures are appropriate for use in axSpA. This qualitative interview study was therefore designed to better understand the patient experience of axSpA and evaluate the content validity of these customized PROMIS^®^ Short Forms to support their use as endpoints in axSpA clinical trials.

## Methods

### Study design

This was a non-interventional, cross-sectional, semi-structured, qualitative concept elicitation (CE) and cognitive debriefing (CD) interview study. Ninety-minute interviews were conducted via telephone with participants from the United States (US) and Germany.

### Participants

Participants with a range of treatment histories were recruited through clinicians by a third-party recruitment agency. All participants provided written, informed consent prior to admission to the study. Participants were required to have a documented diagnosis of axSpA provided by their clinician, be aged ≥ 18 years old, be fluent in English (US participants) or German (German participants) and able to attend and participate in a 90-min interview via telephone.

Participants also had to meet pre-defined eligibility criteria regarding disease features, including: ASAS classification criteria with chronic back pain (≥ 3 months) with onset before 45 years old; BASDAI score ≥ 4 and back pain ≥ 4 on a 0–10 numerical rating scale in the last three months; objective signs of inflammation, identified by a combination of centrally-read magnetic resonance imaging (MRI) evidence at screening of sacroiliitis as defined by ASAS, and C-reactive protein (CRP) either ≥ 5.0 mg/L (MRI+/CRP+) or CRP < 5.0 mg/L (MRI+/CRP−) or no evidence of sacroiliitis and CRP ≥ 5.0 mg/L (MRI−/CRP+) as evidenced by a health care professional’s review of medical records; and for patients with AS, a sacroiliac X-ray radiograph showing definitive sacroiliac joint structural damage.

Participants were excluded if they had any of the following: evidence of complete ankylosis of the spine; an acute episode of active anterior uveitis at the time of signing consent; difficulty hearing, reading or speaking; active ongoing inflammatory diseases other than axSpA, such as reactive arthritis, synovitis or ankylosing spinal hyperostosis; a condition which affects their ability to understand or take part in the study; participated in a clinical study or any other type of medical research within the past 42 days; undergone any major surgery within the past eight weeks, or had chronic pain not caused by axSpA that requires chronic analgesics or other chronic therapy.

Flexible target quotas for sex, age, race, educational attainment, and type of axSpA were employed to ensure the sample was demographically and clinically diverse (Additional file 1: Table S1).

### Qualitative interviews

Interviews were conducted by trained, experienced researchers. A semi-structured qualitative interview guide (included in Additional file 2) was used to guide discussions during the interviews. The interviewer was flexible in order of questioning, followed the lead of the participant, and asked appropriate follow-up questions when topics of interest arose. Interviews were audio-recorded, anonymized, transcribed verbatim, and translated into English where necessary.

Interviews were conducted in two rounds to allow for interim analysis following the first round, and to provide an opportunity for enhancements to the interview guide to be implemented. As a result of this, the second round of interviews had a greater focus on CD to probe more specifically around concepts identified in the first round and to ensure all the customized PROMIS^®^ Short Forms were comprehensively debriefed.

#### Concept elicitation

The aim of the CE section was to explore participants’ experiences of their condition in an open-ended, unbiased manner. Broad, open-ended questions were first asked to elicit information about symptoms and impact concepts that were important to patients. More focused questions were then used to probe on issues that may not have been mentioned during the interview, to ensure study objectives were met.

Information from the CE section of the study was used to refine a disease-specific conceptual model of axSpA that was developed based on a previously conducted literature review. Importantly, the CE section of the interview was conducted prior to the participant seeing any of the items included in the CD section that followed.

#### Cognitive debriefing

The CD section aimed to explore and confirm the relevance of the customized PROMIS^®^ Short Forms in patients with axSpA. This involved a ‘think-aloud’ exercise in which participants read out loud each instruction, item, and response option for the three customized PROMIS^®^ Short Forms and discussed their decision-making around their answer. Participants were also asked detailed questions about the relevance of the items, response options, and recall period.

Participant understanding of each item, response option, and recall period was also investigated if time allowed. Exploration of item understanding was deprioritized in comparison to item relevance due to the PROMIS^®^ items already having well-established understanding across a wide variety of indications.

Information obtained from the CE and CD sections of the interview was used to evaluate the adequacy of conceptual coverage of the customized PROMIS^®^ Short Forms in assessing pain interference, sleep disturbance, and fatigue in adult participants with axSpA, and to confirm that no key concepts had been overlooked.

### Data analysis

Sociodemographic and clinical characteristics were summarized using descriptive statistics. Verbatim interview transcripts were subject to thematic analysis methods (CE interview data) and framework analysis (CD interview data) [20, 21]. Interviews were coded using Atlas.ti software [22].

Saturation analyses are recommended by the Food and Drug Administration (FDA) to confirm the sample size is adequate to fully explore the concepts of interest [23–25]. To do so, the full sample was divided into four groups of seven participants, and researchers established when participants first spontaneously discussed each concept. Saturation was deemed to have been achieved if no new concepts emerged in the final interview set, thereby indicating that all relevant concepts had been identified and it would be highly unlikely that any other new concepts would be identified through the conduct of more interviews. All symptoms and impact domains emerged in the first two sets of interviews, providing evidence to suggest that the symptom and impact experience of axSpA had been fully explored and no further interviews were required.

Frequency counts and detailed descriptions of each concept domain and sub-concept were also presented, and qualitative analysis was performed to establish if participant subgroups (e.g., axSpA type, country) experience or describe axSpA differently.

## Results

### Participant characteristics

A total of 28 participants were interviewed (AS = 16; nr-axSpA = 12) (Table 1). Participants were from the US (n = 20) and Germany (n = 8), with a mean age of 52.8 years (range 32–79 years), and 16 (57%) of the participants were male. All recruitment quotas were met in the US sample except for educational level (n = 6 versus the quota of ≥ 7 for those who have not completed high school or higher). In the German sample, the age quota for participants aged > 61 years was not met (n = 1 versus quota of ≥ 2), alongside the quotas for non-Caucasian ethnicity (n = 0 versus quota of ≥ 3), and axSpA type (n = 1 participant with nr-axSpA versus quota of 4).

**Table 1:** Patient characteristics

Characteristic	Total sample (N = 28)
Age (years), mean (range)	52.8 (32–79)
Male, n (%)	16 (57)
Current living status, n (%)*	
Living with partner	17 (61)
Living alone	6 (21)
Living with children	5 (18)
Living with other family members	3 (11)
Living with parent(s)	1 (4)
Race, n (%)	
White/Caucasian/European	20 (71)
African American/African Heritage	5 (18)
White Arabic/North African Heritage	1 (4)
Mixed race	1 (4)
Other	1 (4)
Highest level of education, n (%) [German option]	
Some high school (15 to 17) [Secondary school]	12 (43)
College or university degree (2/4 years) [University degree]	5 (18)
Some years of college (17 to 18) [University visit]	5 (18)
Graduate or professional degree [Postgraduate degree]	5 (18)
High school diploma or GED [High school]	1 (4)
Work status, n (%)	
Working full-time	12 (43)
Not working due to axSpA	5 (18)
Retired	5 (18)
Looking for work	2 (7)
Full-time homemaker	1 (4)
Working part-time	1 (4)
Unemployed	1 (4)
Student	1 (4)
Time since diagnosis date^†^ (years), mean (range)	9.5 (0.3–31.3)
Type of axSpA, n (%)	
Ankylosing spondylitis	16 (57)
nr-axSpA	12 (43)
Current BASDAI score, mean (range)	6.4 (4–10)
ASAS features currently being experienced, n (%)	
Inflammatory back pain	27 (96)
Arthritis	18 (64)
Elevated CRP	16 (57)
Good response to NSAIDs	13 (46)
HLA-B27	9 (32)
Crohn’s/colitis	5 (18)
Psoriasis	5 (18)
Enthesitis (heel/elbow)	3 (11)
Dactylitis	3 (11)
Uveitis	2 (7)
Family history of SpA	2 (7)
Patient-reported health in general, n (%)	
Excellent	0 (0)
Good	12 (43)
Fair	12 (43)
Poor	3 (11)
Very poor	1 (4)
Presence of co-morbid conditions, n (%)	
Yes	20 (71)
No	8 (29)
Co-morbid conditions^‡^, n (%)	
Ulcerative colitis	5 (18)
Hypertension	4 (14)
Psoriasis	4 (14)
Depression/anxiety	3 (11)
Gastroesophageal reflux disease	2 (7)
Asthma	2 (7)
Migraine with/without aura	2 (7)

^*^More than one option was selected by some participants

^†^Two participants had missing data for time since diagnosis date

^‡^Comorbidities with n = 1 are not reported

*ASAS* Assessment of Spondylarthritis international Society, *axSpA* axial spondyloarthritis, *BASDAI* Bath Ankylosing Spondylitis Disease Activity Index, *CRP* C-reactive protein, *HLA‑B27* human leukocyte antigen B27, *nr* non-radiographic, *NSAID* non-steroidal anti-inflammatory drug, *SpA* spondylarthritis

Mean time since axSpA diagnosis was 9.5 years (range 0.3–31.3 years). The most common spondyloarthritis features from the ASAS classification criteria included inflammatory back pain (n = 27/28, 96%), and arthritis (n = 18/28, 64%). Comorbidities were reported by 20 (71%) participants, the most common of which were ulcerative colitis (18%), hypertension (14%), and psoriasis (14%).

**Fig. 1:**
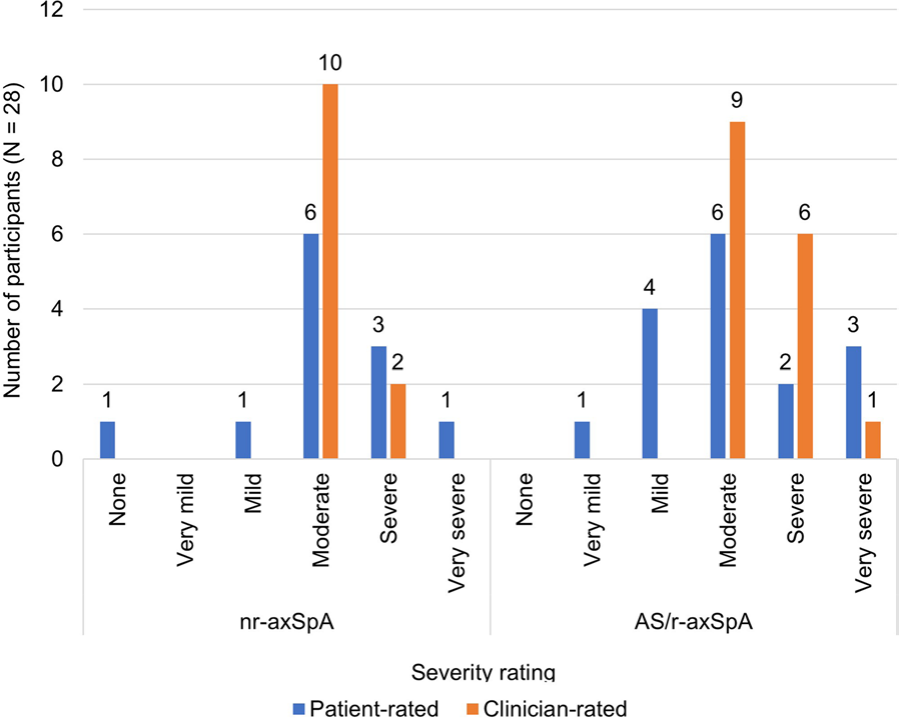
Patient and clinician ratings of axSpA severity. Patients rated disease severity over the past day; clinicians rated severity based on all clinical evidence. *AS* ankylosing spondylitis, *axSpA* axial spondyloarthritis, *nr-axSpA* non-radiographic axial spondyloarthritis, *r-axSpA* radiographic axial spondyloarthritis

Most participants reported their health was generally good (43%) or fair (43%). When asked to rate the severity of their axSpA in the past day, most participants reported their condition as moderate. Clinician ratings of disease severity (based on all clinical evidence) were consistent with patient ratings (Fig. 1).

No substantial differences were observed between patients with nr-axSpA and AS throughout this study; therefore, the following CE and CD sections primarily report pooled results from the overall axSpA sample.

### Concept elicitation

#### Interviews

**Fig. 2:**
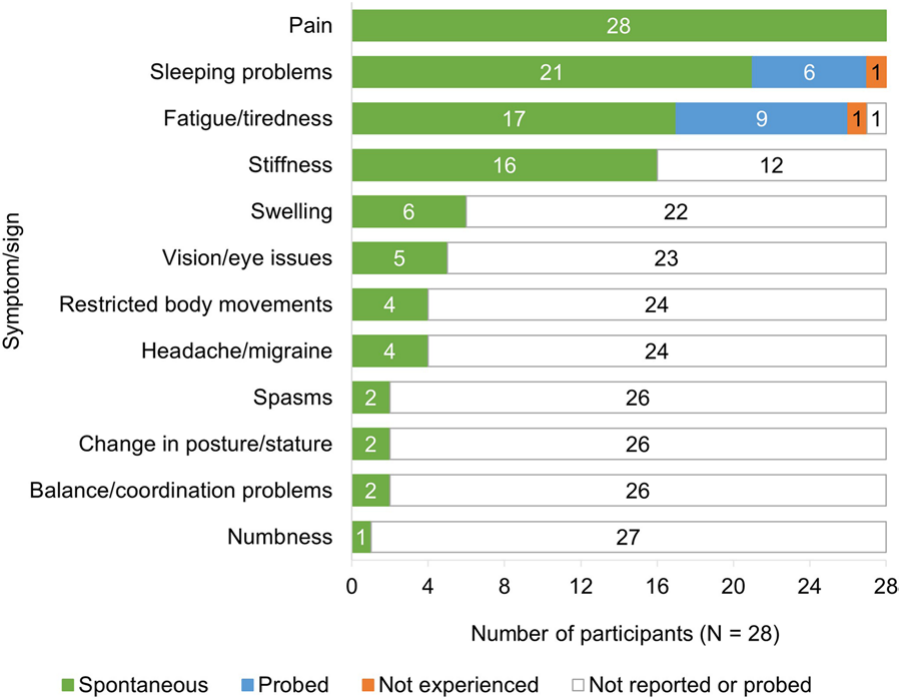
Symptoms/signs of axSpA as reported during concept elicitation. *axSpA* axial spondyloarthritis

A total of 12 distinct symptoms were spontaneously elicited by participants during the interviews: pain, sleep problems, fatigue/tiredness, stiffness, swelling, vision/eye issues, restricted body movements, headache/migraine, spasms, change in posture/stature, balance/coordination problems and numbness (Fig. 2; key quotes included in Additional file 1: Table S2). Pain, sleep problems and fatigue/tiredness were all reported by ≥ 90% of participants as symptoms which occur simultaneously and exacerbate one another.

Pain was spontaneously reported by all participants (n = 28/28, 100%) and was most commonly reported as the first symptom experienced in axSpA (n = 18/28, 64%), and the symptom which first led participants to seek advice from a healthcare practitioner (n = 17/28, 61%). Pain was reported by participants as either continuous (n = 16/28, 57%) or occurring in intermittent episodes (n = 10/28, 36%). Two participants (n = 2/28, 7%) did not comment on whether their pain was continuous or intermittent. Additionally, participants reported that they experienced pain daily (n = 20/28, 71%). Over half of participants (n = 19/28, 68%) reported pain as the symptom they most wished to see improvement in.

Sleep problems due to axSpA were reported by nearly all (n = 27/28, 96%) participants (spontaneously by 21 participants, and by six participants when probed). Most commonly reported sleep problems were difficulty staying asleep (n = 22/27, 81%) and restless sleep (n = 16/27, 59%). Participants described their sleep problems as occurring several times per week (n = 7/27, 26%), every night (n = 4/27, 15%), most nights, quite often, a few days per month, and once per month (n = 1/27; 4% each). Twelve participants did not report how frequently they experienced sleep problems (n = 12/27, 44%). Pain was the most common factor associated with sleep problems, reported by eight participants (n = 8/27, 30%).

Fatigue/tiredness was reported as a symptom of axSpA by 26 participants (n = 26/28, 93%, reported spontaneously by 17 participants and upon probing by nine participants). Participants reported experiencing fatigue daily (n = 8/26, 31%), once or twice a week (n = 6/26, 23%), and periodically (n = 4/26, 15%). Eight participants did not report how frequently they experienced fatigue/tiredness (n = 8/26, 31%). Of the participants who described the duration of their fatigue (n = 10/26, 39%), over half described their fatigue as occurring in intermittent episodes/flare-ups (n = 6/10, 60%). The remaining participants (n = 4/10, 40%) described fatigue as occurring continuously. Fatigue/tiredness was discussed as being linked to pain and sleep problems by nine participants (n = 9/26, 35%).

Stiffness was spontaneously reported as a symptom of axSpA by 16 participants (n = 16/28, 57%). Among participants who reported how frequently they experienced stiffness, stiffness was described as occurring every morning (n = 3/4, 75%) or reoccurring (n = 1/4, 25%). In terms of duration, stiffness was described as lasting for minutes (n = 2/4; 50%), hours (n = 1/4; 25%), or occurring continuously (n = 1/4; 25%). Over half of the participants who reported stiffness described it as a contributing factor to their pain (n = 9/16, 32%). Stiffness was reported to be one of the most bothersome symptoms by a quarter of the participants (n = 7/28, 25%).

Swelling, vision/eye issues, restricted body movements, headache/migraine, spasms, change in posture/stature, balance/coordination problems and numbness were reported by fewer participants (all n ≤ 6/28).

**Fig. 3:**
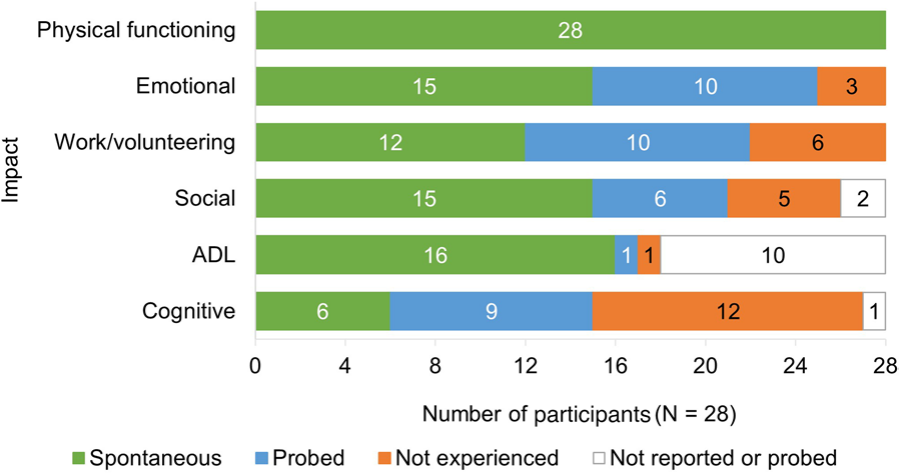
Impacts on HRQoL due to axSpA. *ADL* activities of daily living, *axSpA* axial spondyloarthritis, *HRQoL* health-related quality of life

Participants reported axSpA to impact their lives across six health-related quality of life (HRQoL) domains: physical functioning (n = 28/28, 100%), emotional wellbeing (n = 25/28, 89%), work/volunteering (n = 22/28, 79%), social functioning (n = 21/28, 75%), activities of daily living (n = 17/28, 61%) and cognitive functioning (n = 15/28, 54%) (Fig. 3; key quotes included in Table S3 in Additional file 1). Impacts were most frequently described as being associated with pain, stiffness, and fatigue.

#### Conceptual model

**Fig. 4:**
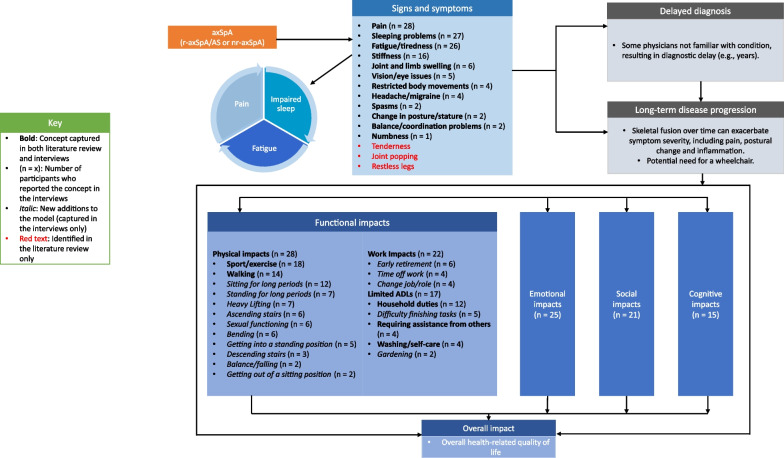
axSpA conceptual model. *ADL* activities of daily living, *AS* ankylosing spondylitis, *axSpA* axial spondyloarthritis, *nr-axSpA* non-radiographic axial spondyloarthritis, *r-axSpA* radiographic axial spondyloarthritis, *r-axSpA* radiographic axial spondyloarthritis

A targeted qualitative literature review was conducted prior to this study to identify key symptoms and HRQoL impacts on patients with axSpA. Concepts identified from the CE interviews were combined with concepts identified in this literature review to form a conceptual model of axSpA (Fig. 4). A total of 15 key signs and symptoms of axSpA were identified through the literature review and qualitative interviews. The interviews confirmed pain, sleep problems and fatigue/tiredness were the most commonly experienced symptoms of axSpA, and stiffness was also highlighted as a bothersome symptom. In addition, the interviews highlighted the greater importance of the different ways in which axSpA impacts participants, both physically and while at work. Most notably, 13 new physical functioning impacts and 6 work/volunteering impacts were added to the revised conceptual model.

### Cognitive debriefing

#### Understanding

**Fig. 5:**
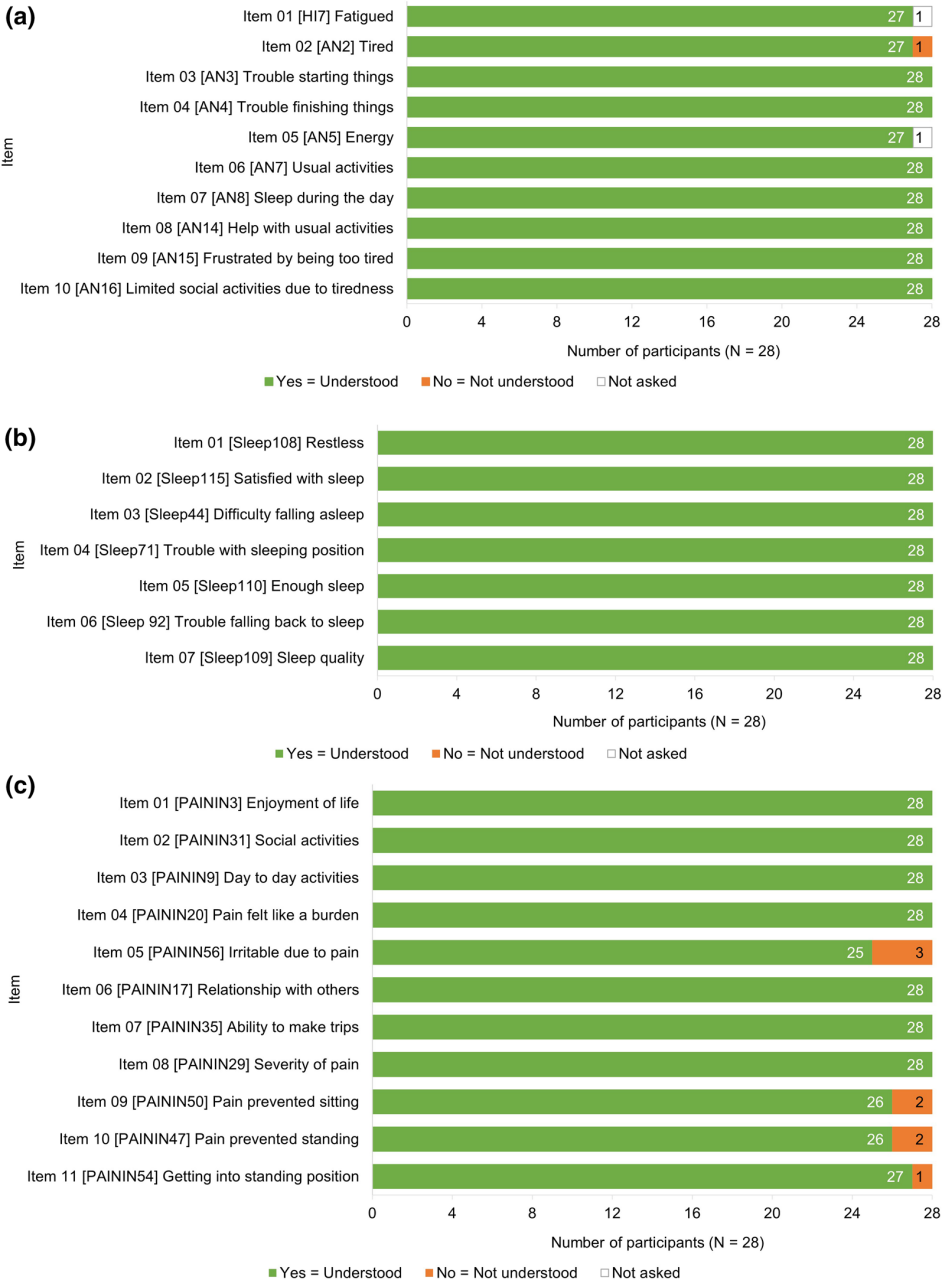
Participant understanding of the customized PROMIS^®^ Short Forms. **A** PROMIS^®^ Fatigue. **B** PROMIS^®^ Sleep Disturbance. **C** PROMIS^®^ Pain Interference. *PROMIS*^®^ Patient-Reported Outcomes Measurement Information System

CD showed all three customized PROMIS^®^ instruments were well understood by patients with axSpA (Fig. 5). One participant (n = 1/28, 4%) had difficulty differentiating between ‘tired’ and ‘fatigued’ in the PROMIS^®^ Fatigue item 02 [AN2]: *Tired*. No participants reported misunderstanding of instructions and/or items of the PROMIS^®^ Sleep Disturbance instrument. All items of PROMIS^®^ Pain Interference were well understood by all participants, with the exception of four items that had a few instances of misunderstanding. Item 05 [PAININ56]: *Irritable due to pain* was not understood by three participants (n = 3/28, 11%) as they had difficulty differentiating between irritability due to pain and irritability due to other reasons. Two participants (n = 2/28, 7%) stated that their answer to item 09 [PAININ50]: *Pain prevented sitting* would differ depending on what they were sitting on. Two participants (n = 2/28, 7%) could not answer item 10 [PAININ47]: *Pain prevented standing* as they could not differentiate between standing still and standing while moving. Item 11 [PAININ54]: *Getting into standing position* was misunderstood by one participant (n = 1/28, 4%) as they did not understand what the question meant regarding not being able to stand and the association with pain.

Probes for recall period understanding were asked for select items of the customized PROMIS^®^ instruments due to interview time constraints. Overall, the seven-day recall period was well understood across the customized PROMIS^®^ instruments. Instances of misunderstanding included references to a different recall period (PROMIS^®^ Fatigue Item 01 [HI7]: *Fatigued,* n = 6/26, 23%; PROMIS^®^ Fatigue Item 10 [AN16]: *Limited activities due to tiredness,* n = 4/20, 20%; PROMIS^®^ Sleep Disturbance Item 02 [Sleep115]: *Satisfied with sleep*, n = 5/24, 21%; PROMIS^®^ Sleep Disturbance Item 07 [Sleep109]: *Sleep quality,* n = 1/26, 4%; PROMIS^®^ Pain Interference [PAININ20]: *Pain felt like a burden,* n = 6/28. 21%). Additionally, a small number of participants (n ≤ 3 per item asked) suggested extending the recall period to capture variation in symptoms.

#### Conceptual relevance

**Fig. 6:**
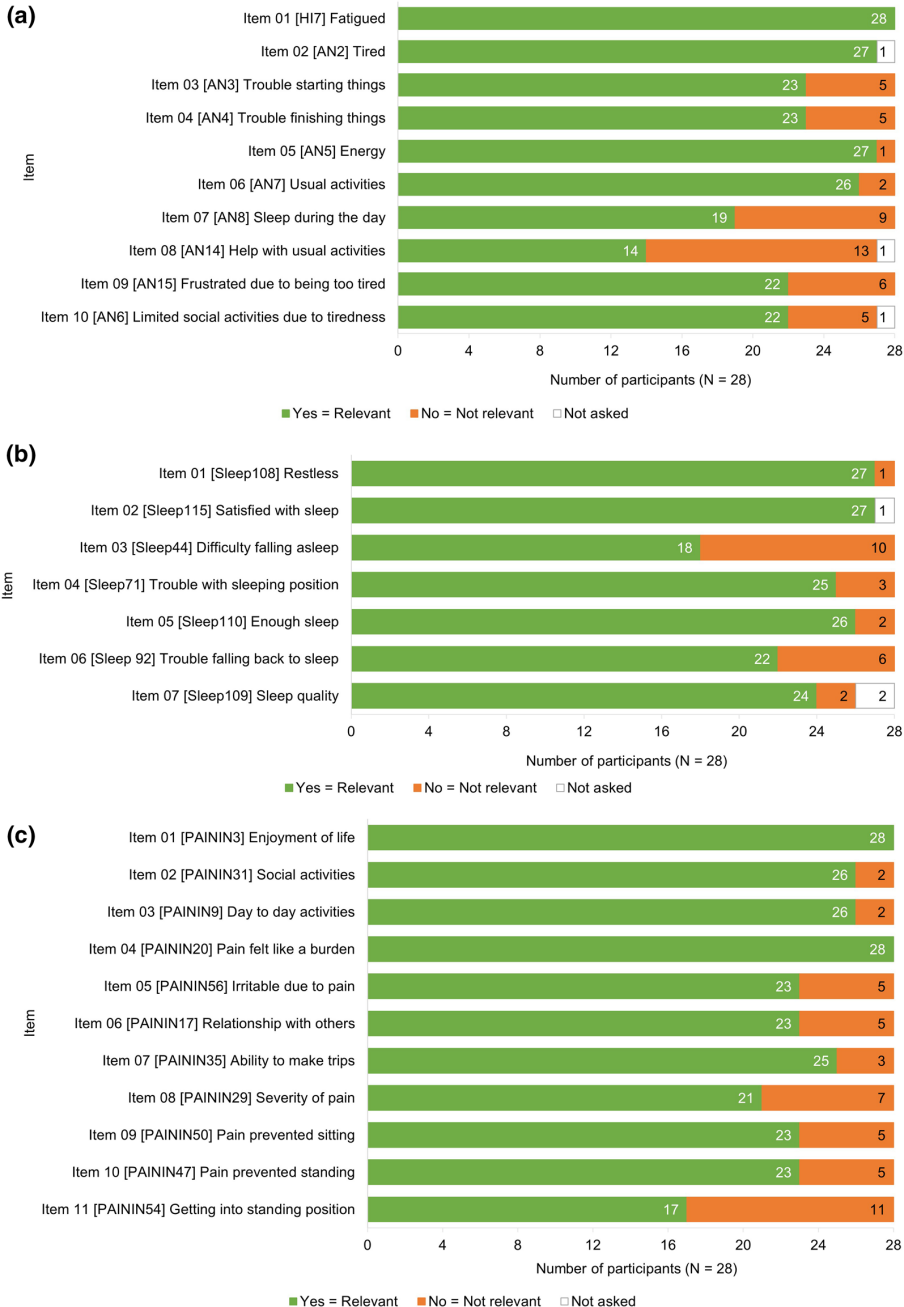
Conceptual relevance of the customized PROMIS^®^ Short Forms. **A** PROMIS^®^ Fatigue. **B** PROMIS^®^ Sleep Disturbance. **C** PROMIS^®^ Pain Interference. *PROMIS*^®^ Patient-Reported Outcomes Measurement Information System

CD showed all three PROMIS^®^ instruments are conceptually comprehensive to patients with axSpA (Fig. 6). Across each instrument, all items were relevant to at least half of participants, and almost all reported the instruments to be appropriate for measuring their experience of sleep problems, pain, and fatigue due to axSpA.

All items of PROMIS^®^ Fatigue were relevant to ≥ 79% of participants (n = 22/28), with the exception of item 08 [AN14]: *Help with usual activities*, and item 07 [AN8]: *Sleep during the day* (reported as not relevant by 46% [n = 13/28] and 32% [n = 9/28], respectively). Participants with moderate axSpA were less likely to report these items as relevant to their disease experience than those with severe axSpA (item 08 [AN14] reported as not relevant by 54% [n = 7/13], 38% [n = 5/13], and 8% [n = 1/13] of participants with moderate, severe, and very severe disease, respectively, and item 07 [AN8] by 78% [n = 7/9] and 22% [n = 2/9] participants with moderate and severe disease, respectively). This pattern was not observed for any of the other PROMIS^®^ Fatigue items.

The majority of PROMIS^®^ Sleep Disturbance items were relevant to all participants. Compared with other items, a higher proportion of participants reported item 03 [Sleep44]: *Difficulty falling asleep* and item 06 [Sleep92]: *Trouble falling back to sleep* as not relevant to their disease experience (n = 10/28 and n = 6/28, respectively).

All PROMIS^®^ Pain Interference items were reported to be relevant by ≥ 82% of the sample (n = 23/28), with the exception of item 08 [PAININ29]: *Severity of pain* and item 11 [PAININ54]: *Getting into a standing position* (reported as not relevant by 25% [n = 7/28] and 39% [n = 11/28] of participants, respectively). There was a slight trend for more participants with AS to report Pain Interference items as not relevant compared to participants with nr-axSpA.

Participants with both AS and nr-axSpA confirmed the three PROMIS^®^ instruments were relevant and appropriate for assessing their disease experience.

## Discussion

This qualitative interview study found that pain, sleep problems and fatigue are core symptoms of axSpA that exacerbate each other and affect HRQoL across six key domains. In addition, the content validity of the three customized PROMIS^®^ Short Forms was evaluated and confirmed. Results were consistently similar between patients with nr-axSpA and AS.

Pain was the most commonly reported symptom of axSpA, experienced by all patients (n = 28/28). It should be noted that this study selected patients who experienced back pain ≥ 4 on a 0–10 numerical rating scale in the last three months; therefore, pain was expected to be experienced by this sample. However, pain is well recognized as a key symptom for patients with axSpA across the literature [26, 27].

Appropriate PRO instruments are necessary to quantify the symptom burden of axSpA, and for the investigation of treatment benefits. There are currently limited high-quality, comprehensive PRO instruments for use among the axSpA population [28]. Existing PRO instruments for axSpA do not conceptually cover all key measurement concepts and associated impacts, which is what the three customized PROMIS^®^ Short Forms aim to do.

This study broadly confirms the interpretability, comprehensiveness and, therefore, content validity and suitability of the three customized PROMIS^®^ Short Forms in clinical trials in axSpA. Across all three instruments, all items were relevant to at least half of the sample, and only two items had particularly lower relevance than the others (PROMIS^®^ Pain Interference item 11 [PAININ11]: *Getting into a standing position* and PROMIS^®^ Fatigue item 08 [AN14]: *Help with usual activities*). Instances of items being reported as not relevant to the disease experience by a small number of participants is expected, given each individual’s unique experience of axSpA and varying levels of disease severity. There appeared to be a slight pattern within the PROMIS^®^ Pain Interference items whereby more participants with AS reported the items as not relevant compared with participants with nr-axSpA, indicating that some items may only be relevant to patients with nr-axSpA. As there was no clear pattern in severity for the PROMIS^®^ Fatigue and PROMIS^®^ Sleep Disturbance items, these nuances were likely a result of unique experiences of axSpA to each individual or the clinical characteristic make-up of the sample. For example, the sample contained more participants with disease of moderate severity than severe disease.

Although very few, there were some instances of misunderstanding, or participants not finding items relevant. This is expected due to the heterogenous nature of axSpA and the individuality of the patient experience. It should be noted that misunderstanding among the German sample was not believed to be due to translation issues, as translations were certified by the developer and the reasons participants provided for misunderstanding of items appeared to be unrelated to such issues. To overcome instances of misunderstanding, patient training could be carried out prior to first completion of the customized PROMIS^®^ Short Forms, to ensure that participants fully understand each instruction, item, and response option to reduce the chance of incorrect completions.

Although the recall period was well understood across the sample, a small number of participants suggested that the seven-day recall period should be adjusted to capture periods of ‘flare-ups’. It is therefore recommended that individuals who are considering implementing the customized PROMIS^®^ instruments within clinical trials consider the timepoints of administration to accurately capture ‘flare-ups’. However, with a large enough sample, it would be expected that a certain percentage of participants would be experiencing a ‘flare-up’ at any one time, and the recall period is therefore less of a concern.

These findings support the use of customized PROMIS^®^ Pain Interference, Fatigue, and Sleep Disturbance Short Forms to assess the impact of axSpA on patients. However, some of the physical functioning, stiffness and broader impacts on work are not covered by the three PROMIS^®^ instruments. The combination of these customized PROMIS^®^ Short Forms with other PRO assessments that measure physical functioning, stiffness, and impacts on work (such as BASDAI, BASFI and WPAI) would therefore allow for a comprehensive assessment of the patient experience of axSpA and the benefit of treatment interventions. Psychometric validation of the PROMIS^®^ instruments in the target population should be conducted to confirm their reliability and validity.

### Limitations

Due to the qualitative nature of the study, there were limitations to the sample size. However, the overall sample size was judged to be adequate based on the saturation analysis. Consequently, all concepts identified as important in the literature review were captured and it is likely that little to no new information would be elicited with the conduct of further interviews [24, 29, 30]. In addition, sample quotas were employed per country to ensure a diverse sample and reflection of each demographic and clinical characteristic. The US quota for participants who had not completed high school was not achieved and, as a result, items which were more difficult to understand, or used complex language, may not have been identified by this sample. Despite this, the sample appeared to reflect the broader axSpA population in regard to age and sex. Due to the small sample size in Germany (n = 8), a comparison of results between the US and Germany was not appropriate. Interviews were 90 min long, which may have induced fatigue effects. Participants were offered a break halfway through to address possible fatigue effects and, in the second round of interviews, the order of the customized PROMIS^®^ Short Forms was changed to avoid higher numbers of misunderstood/non-relevant items as a result of participants becoming disengaged. It must also be noted that the customized PROMIS^®^ Pain Interference Short Form measures pain generally and, therefore, if it is important to measure a specific type of pain associated with axSpA (e.g., joint/spine pain or uveitis), then more pain-specific measures should be considered. Finally, four participants had recently experienced COVID-19, which may have affected these participants’ daily routines and/or experiences of living with axSpA.

## Conclusions

Pain, sleep problems and fatigue are pivotal symptoms of nr-axSpA and AS classifications of axSpA and are associated with HRQoL impacts. Interpretability and content validity of the customized PROMIS^®^ Short Forms have been confirmed, with each deemed to adequately assess key impacts associated with axSpA, supporting their potential future use in clinical trials of patients with axSpA.

## Supplementary Information

**Additional file 1.** Sampling quotas and key patient quotes.

**Additional file 2.** Interview guides.

## Data Availability

The datasets generated and/or analysed during the current study are not publicly available as, although transcripts were anonymized, participants could be identified from an entire dataset.

## Abbreviations

ADL: Activities of daily living
AS: Ankylosing spondylitis
ASAS: Assessment of SpondyloArthritis international Society
ASQOL: Ankylosing Spondylitis Quality of Life
axSpA: Axial spondyloarthritis
BASDAI: Bath Ankylosing Spondylitis Disease Activity Index
BASFI: Bath Ankylosing Spondylitis Functional Index
CD: Cognitive debriefing
CE: Concept elicitation
COVID-19: Coronavirus disease 2019
CRP: C-reactive protein
EQ-5D: EuroQol 5 Dimensions
FDA: Food and Drug Administration
HLA‑B27: Human leukocyte antigen B27
HRQoL: Health-related quality of life
MRI: Magnetic resonance imaging
nr: Non-radiographic
nr-axSpA: Non-radiographic axial spondyloarthritis
NSAID: Non-steroidal anti-inflammatory drug
PRO: Patient-reported outcome
PROMIS^®^: Patient-Reported Outcomes Measurement Information System
RA: Rheumatoid arthritis
r-axSpA: Radiographic axial spondyloarthritis
SF-36: 36-Item Short Form Survey
SpA: Spondylarthritis
US: United States
WPAI-axSpA: Work Productivity and Activity Impairment Questionnaire axial spondyloarthritis
