# Supplementary material for: Qualitative concept elicitation and cognitive debriefing interviews of symptoms, impacts and selected customized PROMIS® Short Forms: a study in patients with axial spondyloarthritis
Source: J Patient Rep Outcomes. 2023 Apr 20;7:39. doi: 10.1186/s41687-023-00575-x (PMC10117270; doi:10.1186/s41687-023-00575-x)
Supplement: Supplementary file 1 — Additional file 1. Sampling quotas and key patient quotes. [file 41687_2023_575_MOESM1_ESM.docx]

# Supplement 1

**Supplementary Table 1** Sampling quotas used in participant recruitment

| **Participant characteristics** | | **US (N=20)** | **Germany (N=8)** |
| --- | --- | --- | --- |
|  |  | **Quota** | **Quota** |
|  |  |  |  |
| Sex | Male | ≥7 | ≥3 |
|  | Female | ≥7 | ≥3 |
| Age (years) | 18–40 | ≥5 | ≥2 |
|  | 41–60 | ≥5 | ≥2 |
|  | 61+ | ≥5 | ≥2 |
| Race | Caucasian/White | ≥7 | ≥3 |
|  | Non-Caucasian/White | ≥7 | ≥3 |
| Educational attainment | Has completed high school or higher | ≥7 | ≥3 |
|  | Has not completed high school or higher | ≥7 | ≥3 |
| Type of axSpA | AS | 10 | 4 |
|  | nr-axSpA | 10 | 4 |

*AS* ankylosing spondylitis, *axSpA* axial spondyloarthritis, *nr-axSpA* non-radiographic axial spondyloarthritis, *US* United States

**Supplementary Table 2** Key patient quotes – signs/symptoms of axSpA (CE section)

| **Description** | **Supporting quotes** |
| --- | --- |
| **Pain** | |
| Sharp pain (reported by 25% participants [n = 7/28]) | *“Um, ah, so depending on the joint, it might be a sharp aching pain. Um, in particular, um, my knees tend to have more of sharp like pains in them.”* |
| Aching pain (reported by 18% participants [n = 5/28]) | *“…it's kind of like a dull ache in the back and, and shoulders...”* |
| Dull pain (reported by 18% participants [n = 5/28]) | *“You know, I think it differs. It could be anything from sharp pains to, you know, dull* *aches.”* |
| Severity (via interviewer-administered 0–10 numeric rating scale) | Bad day:  “*Horrible, will be almost 10.”*  Good day:  *“Well the pain is always there. I could tell you that. If we want to say from one to ten, it's always there like between five and six.”* |
| Duration | *“I always have in the back of my mind ‘there it is again, you have the pain again’ and this restricts you to an extent even though the restriction are not that bad yet. It’s just always present.”* |
| Change over time | *“And, um, not only did the, um, the pain start to increase over time. I started to develop more fatigue and, uh, some days just kind of felt like I was in a fog.”* |
| **Sleeping problems** | |
| Difficulty staying asleep (reported by 81% participants [n = 22/27]) | “*Uh, difficulties, yeah, staying asleep. There's times where I, I have to get up and kind of walk around and, you know, just—I'll do some stretching and before trying to go back to sleep.*” |
| Restless sleep (reported by 59% participants [n = 16/27]) | “*When it's acute, I have problems to fall [sic] asleep, as I don't find a good sleeping position.* *My sleep is restless and [I] wake up often as I feel pain. The next day I feel very unrelaxed and unbalanced, of course*.” |
| Sleep quality (reported by 48% participants [n = 13/27]) | “*A bad day is when you get up in the morning and the whole back hurts, you feel a little out of it because you probably didn’t sleep well during the night and you tossed and turned. It is difficult to get going and the pain remains for longer.*” |
| Severity (via interviewer-administered 0–10 numeric rating scale) | Bad day: *“Um, I'll typically—when I wake up from pain, you know, at the most I'm able—I'm ever up from it would be for two hours. But much more often I'm up for half an hour or an hour and then I'm able to fall back to sleep. There are sometimes when that happens twice in the night, but I—so I think I would say, um, you know, six, six or seven.”*  Good day:  *“Um, I would say on a, on a good day, um, I'd go with, you know, maybe a one. It might take me a little bit to get to sleep, but, but I'll have a good, you know, a good sleep.”* |
| Duration | “*…And I'm talking just the most mild, like waking up for an hour or half an hour or something, not terrible.*” |
| Change over time | “*…Well, it was really horrible in the beginning until the stiffening finally set in properly and the pain wasn’t there anymore*.” |
| **Fatigue/tiredness** | |
| Tiredness (reported by 19% participants [n = 5/26]) | “*It is just more tiredness. You just want to lie down and just sleep.*” |
| Fatigue (reported by 19% participants [n = 5/26]) | *“I mean definitely the fatigue is one of the more noticeable symptoms for me.*’’  “*…fatigue, it, it, it really, it comes in, in waves and it comes very quickly and it can just feel, feel like a tidal wave.*” |
| Exhaustion (reported by 15% participants [n = 4/26]) | “*When there already is pain, then it certainly worsens and if not, then … No, exhaustion certainly has an impact on it.*”  “*Well, I think I’m exhausted because of the pain and I’m tired of it, and sometimes I will do like a nap during the day.*” |
| Severity (via interviewer-administered 0–10 numeric rating scale) | Bad day: *“I would say on a bad day it's about, um, an eight, um, because it'll be just, uh, very hard for me to get up…. it's just taking forever for me to do these basic things…”*  Good day:  *“Um, on a good day, you know, maybe like two or three.”* |
| Duration | “*Yes, and I am actually tired the whole day. It is basically like I did not sleep at all.”*  “*About half an hour I would say and then the worst is over. I would sit down somewhere and do nothing for half an hour, then you get back to normal and you have some energy again.”* |
| Change over time | “*…the symptoms that have progressed [worsened] for me… are fatigue and depression*.”  “*Now after a year, also with the drugs I am receiving, I notice that it is improving a little bit.”* |
| **Stiffness** | |
| Severity (via interviewer-administered 0–10 numeric rating scale) | Bad day:  *“Um, for the spasms I would say a bad day would probably be, uh, a seven”*  Good day:  *“Now that on a, on a very good day as far as the stiffness, I would put it at a* ***zero*** *because that's how I feel, you know, I feel whole.”* |
| Duration | *“I get up and have this stiffness in the morning for 10 minutes at the most.”*  *“Throughout the day.”* |
| **Additional symptoms** | |
| Swelling | “*Um, joints swollen, you know. Couldn’t get, couldn’t get a ring on, you know, um, and just gripping, gripping tools, gripping, gripping even a fork can be difficult. But I again push through*.” |
| Vision/eye issues | *“…the constant blurry vision, sensitive to light, and then your eyes just kill you and you get a monster headache after that, yeah. It just—but, you know, that comes along with pain.”* |
| Headache/migraine | *“If I don't do anything about it, I get severe headaches. Then I'm sensitive to touch in certain joints in my hand and feet. Standing is, of course, difficult then, because every type of movement increases the pain.”* |
| Restricted body movements | *“And, um, it just was really hard to do things. You just weren't as flexible anymore.”* |
| Spasms | *“The spasms for me, um, it, it tends to be like a harbinger for me of, of the pain. Sometimes, uh, the spasms come just before the pain comes…”* |
| Change in posture/stature | *“Then, of course, shoulder, chest area, there's always tension and, and therefore also pain, because of the bad posture I have.”* |
| Balance/coordination | *“If I sit for very long, I get up, it's very difficult to start the movement process of walking, unsteady on the feet.”* |
| Numbness | *“I mean I definitely feel like a dull pain, um, in my back and, uh, shoulders…”* |

*axSpA* axial spondyloarthritis, *CE* concept elicitation

**Supplementary Table 3** Key patient quotes – impacts of axSpA on HRQoL (CE section)

| **Description** | **Supporting quotes** |
| --- | --- |
| **Physical functioning** | |
| Sport/exercise (reported by 64% participants [n = 18/28]) | “*So, it started to limit me, since I wasn’t able to do certain exercises in sports that well anymore, or I knew that it would cause me quite some trouble the next day…* *in the end I couldn’t bear it for one minute, because my shoulders were burning.*” |
| Walking (reported by 50% participants [n = 14/28]) | *“…So if I'm having a bad day and I, and I know that if I take a long walk it's going to make it even worse, I will skip a day of taking a walk. So I do—so depending on how I feel, I will avoid activities because, uh, I'm afraid I'll contribute to the pain.”* |
| Sitting for long periods (reported by 43% participants [n = 12/28]) | “*…The only thing we also don’t do anymore because of it is, um,* *going to the theater or so, because, um, I can’t sit for long*.” |
| Standing for long periods (reported by 25% participants [n = 7/28]) | “*I will make some, you know, space in home where I can do the things by sitting down, you know, like prepare for the, you know, dinner or other stuff, you know, writing or, ah, standing don't help me at all. It’s actually getting me more in pain*.” |
| Heavy lifting (reported by 25% participants [n = 7/28]) | “*Um, carrying heavy objects for example or helping friends to move, where you also carry heavy loads. This is just not possible because it really hurts.”* |
| **Emotional wellbeing** | |
| Depression/sadness (reported by 68% participants [n = 17/25]) | “*…You don’t realize, you know, what a job actually does for you as far as, you know, you know, giving you, you know, that, that sense of, you know, that sense of purpose and, you know, self-worth, stuff like that. You know, that can take a toll on you and that adds on to the depression*.”  *“Well it, it kind of puts you in a position you don’t really want to go do a lot of activities. Um, so you miss out on, you know, things that if, you know, at the time when I was feeling healthy that I would normally do.”* |
| Frustration (reported by 24% participants [n = 6/25]) | “*…I think it does in the sense of frustration. Like, uh, you know, like you're in so much pain you can't really think. Like sometimes I find myself at work…I lose concentration. I lose focus with this disease*.” |
| Anxiety/worry (reported by 12% participants [n = 3/25]) | “*I be like, damn, I don’t like the way it looks, you know, the way I look with, with my neck hanging over like that. Yeah. It affects me emotionally. It gives me anxiety. Sometimes I get anxiety attacks…”* |
| Fear (reported by 12% participants [n = 3/25]) | “*At that point it was quite negative, because you said to yourself, Jesus, now I’ll get a crooked back and I will run around bent forward. That was the biggest fear actually*.” |
| **Work/volunteering** | |
| Early retirement (reported by 27% participants [n = 6/22]) | *“The greatest change was that I couldn't work, anymore. I asked myself: Are you still important and valuable?”* |
| Time off work (reported by 18% participants [n = 4/22]) | *“I would have to take, you know, sick days and, um, so for many years* *I really had very few sick days, you know, where I would just have maybe one or two sick days left to take. Uh, but because—being able to work from home and getting some extra rest on those days, um, I'm—that's made a difference with that where I've had—now I have, uh, a fair amount of sick leaves.”* |
| Change job/role (reported by 18% participants [n = 4/22]) | *“So like we did a pirate ship for a parade float here and it was like a full scale ship that I went and did all the detail, all the gold work, all the, you know, the color and everything. I can't do that, that type of thing anymore...”* |
| **Social functioning and relationship** | |
| Issues with intimate relationships (reported by 38% participants [n = 8/21]) | “*I don’t know how much you want me to get into this 'cause it, it can affect a lot of different things. You know, relationships. I mean it can screw around with your life a lot.*” |
| Ability to make social trips with others (reported by 33% [n = 7/21]) | “*…And it really sucks for trying to make plans too. You try to make plans to do something and it could be the day before and you plan for months and then all of the sudden, you know, you get a flare-up, right. So that's another way it affects you. Like it's hard to make plans. You can't really—you know, it's hard to plan for anything kind of in the future.*” |
| Unable to attend social events (reported by 33% participants [n = 7/21]) | “*…And as I said, with people, to meet up, not thinking of Corona [virus], where this is limited anyway… But there were times without such strict restrictions and then I also completely withdrew, also because I* *can’t sit down for long with my pain.”* |
| Leisure activities (reported by 24% participants [n = 5/21]) | “*…But, uh, in my personal life, as far as on vacations, you know, I don’t swim like I used to. I don’t participate in a snorkelling and, you know, all the activities that we do when we go on like a beach vacation*.” |
| **Activities of daily living** | |
| Household duties (reported by 71% participants [n = 12/17]) | *“It's hard for me to properly clean my flat, when spring cleaning is necessary. In the past I used to do this without any breaks with my wife. Now I need to take breaks, as I can't do certain movements anymore or need to find alternative ways of doing them…”* |
| Difficulty finishing tasks (reported by 29% participants [n = 5/17]) | *“I'm having more trouble doing big projects like finishing—like my den, I'm a little embarrassed, the flooring is not finished in that. There's actually—it's just like the bare plywood right now because I need to finish putting the hardwood flooring in and I just haven't felt up to it…”* |
| Requiring assistance from others (reported by 24% participants [n = 4/17]) | “*But when you really have to drag on and say, “I had to today,” and then I look at the windows and say, “Not today. I’ll do it tomorrow,” or, “I’ll do it the day after tomorrow,” and then that’s that. I am at an age at which I say, “No way. I can still see through that window,” and my husband is helping me making the bed. He does a lot in the house. He is pensioner, he has time. He is taking care of everything and his lovely wife…* *.*” |
| Washing/self-care (reported by 24% participants [n = 4/17]) | *“And, um, then I wash my hair, but it becomes already sketchy at a certain point then, when, for instance, I additionally do some conditioning or whatsoever. Then it takes longer and then I feel that it really starts, um, it really begins do [to] burn then. And then I also have to support myself against the tub from time to time to give some ease to the back for a moment, and then I can, so to speak, build up stability again.”* |
| **Cognitive** | |
| Concentration (reported by 67% participants [n = 10/15]) | *“I would say, it mainly comes from the pain, that you are unable to concentrate… Of course also this constant tiredness, you see? This exhaustion, this physical weakness you have because you hardly sleep and don’t sleep well. Of course this also has an impact on the concentration.”* |
| Motivation to start tasks (reported by 27% participants [n = 4/15]) | *“it's just you, you can't, you can't really do what you want to do at the spur of the moment if you are, you know, really bad with the pain. Um, you are tired. Um, you're tired but you have to move when you're tired. It's terrible.”* |
| Memory (reported by 13% participants [n = 2/15]) | *“…It's like I forget where or what, what I'm at and what am I doing and, you know, what day is this or by the time I get so exhausted.”* |
| Speech problems (reported by 7% participants [n = 1/15]) | *“I would say something, something, and all of a sudden I forgot the word. It’s just right there, it’s just right there and I can’t get out…”* |

*axSpA* axial spondyloarthritis, *CE* concept elicitation, *HRQoL* health-related quality of life
