# Supplementary material for: Qualitative concept elicitation and cognitive debriefing interviews of symptoms, impacts and selected customized PROMIS® Short Forms: a study in patients with axial spondyloarthritis
Source: J Patient Rep Outcomes. 2023 Apr 20;7:39. doi: 10.1186/s41687-023-00575-x (PMC10117270; doi:10.1186/s41687-023-00575-x)
Supplement: Supplementary file 2 — Additional file 2. Interview guides. [file 41687_2023_575_MOESM2_ESM.docx]

# Supplement 2

**Round one interview guide**

# Part 1: Interview introduction (5 minutes)

## Objective

Introduce yourself to the participant, review the purpose of the study and confidentiality arrangements, ask for the participant’s permission to audio record the interview and record your name, the participant’s ID, the date, and the local time and location for you and the participant.

## Interviewer instructions

1. **Thank the participant for agreeing to take part in this interview.**
2. **Introduce yourself as working on behalf of Adelphi Values:**

- My name is [your name] and I am working on behalf of a health research company called Adelphi Values that works closely with pharmaceutical companies to assess the impact of health conditions and treatments on peoples’ lives.

1. **Screening for COVID-19 (ideally, if the participant is not feeling well, he or she should call the interviewer (or recruiter or site?) to reschedule prior to the day of the scheduled interview)**

- Before we start, I need to ask you some questions about COVID-19. I want to make sure that you are feeling well today and able to spend the next 90 minutes with me to help us with our study. [PLEASE NOTE DOWN THE PARTICIPANT’S RESPONSES TO THE FOLLOWING QUESTIONS]

1. **Have you had COVID-19 (either suspected, probable or confirmed)? [Yes or No]**
   1. **If Yes, select the option that best describes you:**

- **Suspected/Probable: I have recently had COVID-19-like symptoms (for example, shortness of breath, cough, fever, extreme fatigue)**
- **Confirmed: I have or have had a confirmed diagnosis of COVID-19**

1. **If answer to 1 is Yes – Have you been symptom-free for the past 14 days? [Yes or No]**
   1. **If answer is No – Schedule the interview to 3 weeks’ time at the earliest**
   2. **If answer is Yes – When did you start experiencing symptoms OR receive your diagnosis of COVID-19? [DD-MMM-YYYY]**
2. **If answer to 1 is No – Are you feeling ill or unwell today? If No – continue with the interview, If Yes – reschedule interview.**
3. **Explain the aim of the interview:**

- The aim of the first part of the interview is to understand your experience of living with your axial spondyloarthritis, including the symptoms and the impact that this has on your life.
- The second part of the interview is designed to get your feedback on three questionnaires which ask about fatigue, sleep and pain. Can you confirm you have copies of the three questionnaires in front of you? You should also have three pieces of paper, each with a large thermometer printed on them; can you confirm you also have these in front of you?

1. **Reassure the participant of confidentiality and anonymity:**

- Your name and contact information will remain with the researchers at Adelphi Values and will only be accessible to staff directly involved with this project.
- Any information you provide will be reported in a way that protects your privacy by avoiding any mention of your name or other information that could identify you.
- The recording of the interview will be typed up, word for word, and will be shared with the sponsor of this project but your name or any information that identifies you will be removed first.

1. **Discuss the interview:**

- The interview will up to 90-minutes.
- The interview will be audio-recorded to allow me to pay careful attention to what you say and to make certain we accurately record the things that you tell me during the interview.
- Please try and speak relatively loudly and clearly so that your comments can be heard and are clear on the recording.
- We want to let you know that the questions during the interview may be repetitive, but that this is necessary, as we want your opinion on the questions and answers.
- You do not have to answer any questions you do not feel comfortable answering.
- You can ask questions at any point in the interview.
- There are no right or wrong answers; we are interested in your personal opinion.
- You can pause or leave the interview at any point.
- You will be compensated after completing the interview.

1. **Turn on audio-recording device *
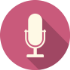
***

- The recording has now begun.
- This is [NAME OF INTERVIEWER] with participant [PARTICIPANT ID NUMBER] on [DATE OF INTERVIEW] at [TIME OF INTERVIEW AND TIME ZONE]. The interview is being conducted by [TELEPHONE/VIDEO]. I am taking the call in [STATE LOCATION OF INTERVIEWER, INCLUDING CITY/STATE AND COUNTRY] and the participant is taking the call in [STATE LOCATION OF PARTICIPANT].
- Do you agree to participate in the interview?
- Do you agree to have this interview audio-recorded?
  - [IF PARTICIPANT DOES NOT AGREE TO HAVE THE INTERVIEW RECORDED: Thank the participant for taking part and end the interview and switch off the recorder.]
- Are you in a location where it is safe to participate in the interview?
- Do you expect to be distracted or have your attention required elsewhere in the next 75-90 minutes?
  - [IF NOT FOR EITHER OF THE ABOVE: Arrange to reschedule the interview and switch off the recorder.]
- Do you have any questions before we start?
- OK, I’ll start now, ok?

# Part 2: Concept elicitation of patient experience of axSpA (35 minutes)

## Objective

The objective of this section of the interview is to encourage the participant to talk spontaneously about the experience of axSpA, including the symptom and treatment experience and how the condition impacts daily life. More probed questions are provided if the participant does not spontaneously discuss all topics of interest. Probes to explore each reported symptom in detail are provided in .

- Today I would like to talk to you about your [axial spondyloarthritis/ ankylosing spondylitis] . When talking about your [axial spondyloarthritis/ ankylosing spondylitis] to your friends and family, what do you call it? What do you refer it as?

TERM(S) USED: ______________________________________

- Ok, so for the rest of the interview, I will refer to your [patient term for axSpA] if that’s ok?

## Symptoms of axSpA

- We are now going to talk about your symptoms and experiences of [patient term for axSpA**].**

1. **Thinking back to when you first started experiencing symptoms associated with your [**patient term for axSpA**], what symptoms did you experience?**
   - W*hat did you first notice that was different?*
   - *Since you first experienced the symptoms, how have they changed until this point, if at all?*
   - *What led you to seek advice from your doctor?*
   - *What did you experience after your diagnosis?*
2. **Please tell me about your day to day experience of having [**patient term for axSpA**].**
   - *What is a typical day like with [patient term for axSpA]*
     - *Is this different to when you were first started experiencing symptoms? How?*
   - *What is a good day like with [patient term for axSpA]*
     - *What do you experience?*
     - *How do you feel?*
   - *What is a bad day like with [patient term for axSpA]*
     - *What do you experience?*
     - *How do you feel?*
     - *How often do you have bad days?*

| **TICK EACH SYMPTOM THAT THE PATIENT MENTIONS SPONTANEOUSLY TO QUESTIONS 1 AND 2 IN THE TRACKING TABLE (Table 1). INSERT ANY ADDITIONAL SYMPTOMS THAT ARE DISCUSSED INTO THE TABLE. ENSURE THE KEY ASPECTS ARE DISCUSSED FOR EACH PROBE BY TICKING THE TOPIC IN THE PROBE COLUMN. IF THE PARTICIPANT DOESN’T SPONTANEOUSLY DISCUSS A KEY TOPIC FOR THAT SYMPTOM (E.G. SEVERITY) THEN PLEASE USE THE SPECIFIED PROBES TO ASK ABOUT THIS.** | | |
| --- | --- | --- |
| Table 1. Symptom tracking table | | |
| Symptom | Tick when mentioned by patient | Symptom-specific probe   - Please tick when information obtained |
| Tiredness and fatigue | □ | - ***Description***   *Tell me more about your tiredness and fatigue.*  *What does this feel like?*  *Could you put that in other words for me?*   - ***Severity***   *Describe how bad or severe the tiredness and fatigue is.*  *How severe would you rate it on a bad day, from 0-10?*  *How severe would you rate it on a good day, from 0-10?*   - ***Frequency***   *How often do you experience tiredness and fatigue with your [patient term for axSpA]?*  *How long does tiredness and fatigue last?*   - ***Triggers***   *Is there anything that triggers the tiredness and fatigue?*   - ***Worsening/improvement***   *Tell me about anything that makes the tiredness and fatigue worse.*  *Tell me about anything that makes the tiredness and figure better.*   - ***Day vs night***   *Is your experience of tiredness and fatigue different during the day versus in the evening before you go to bed (if at all)? How?*  *Is it worse at any particular time of day?*   - ***Change over time***   *Has your tiredness and fatigue changed over time? How?*  *Has a change in tiredness and fatigue been important to you? How? Why?*  Weather/humidity  *Does the weather or humidity affect the tiredness and fatigue in any way? How?* |
| Sleeping problems | □ | - ***Description***   *Tell me more about your sleeping problems.*  *Do you find it hard to go to sleep?*  *Do you have difficulties staying asleep?*  *Do you have difficulties waking up?*   - ***Severity***   *Describe how bad or severe your sleeping problems are.*  *How severe would you rate it on a bad day, from 0-10?*  *How severe would you rate it on a good day, from 0-10?*   - ***Frequency***   *How often do you experience sleeping problems because of your [patient term for axSpA]?*   - ***Triggers***   *Is there anything that triggers your sleeping problems?*   - ***Worsening/improvement***   *Tell me about anything that makes the sleeping problems worse.*  *Tell me about anything that makes the sleeping problems better.*   - ***Change over time***   *Have your sleeping problems changed over time? How?*  *Has a change in your sleeping problems been important to you? How? Why?*   - **Weather/humidity**   *Does the weather or humidity affect your sleeping problems in any way?* |
| Pain | □ | - ***Description***   *Tell me more about the pain you experience due to [patient term for axSpA].*  *What does it feel like?*  *Could you put that in other words for me?*   - ***Location***   *Where do you experience that on your body?*  *Is it always in the same place, or in different places?*  *Do you experience pain in different places on your body at the same time?*   - ***Severity***   *Describe how bad or severe the pain is*  *How severe would you rate it on a bad day, from 0-10?*  *How severe would you rate it on a good day, from 0-10?*   - ***Frequency***   *How often do you experience pain due to your [patient term for axSpA]?*  *How long does the pain you experience last?*   - ***Triggers***   *Is there anything that triggers the pain?*   - ***Worsening/improvement***   *Tell me about anything that makes the pain worse.*  *Tell me about anything that makes the pain better.*   - ***Day vs night***   *Is your experience of your pain different during the day versus when you are in bed/sleeping (if at all)?*  *Is it worse at any particular time of day?*   - ***Change over time***   *Has the pain changed over time? How?*  *Has a change in pain been important to you? How? Why?*   - ***Weather or humidity***   *Does the weather or humidity affect the pain you experience in any way? How?* |
| *(insert any new symptoms)* | □ | - ***Description***   *Tell me more about [symptom]?*  *What does it feel like?*  *Could you put that in other words for me?*   - ***Severity***   *Describe how bad or severe the [symptom] is?*  *How severe would you rate it on a bad day, from 0-10?*  *How severe would you rate it on a good day, from 0-10?* |
| *(insert any new symptoms)* | □ | - ***Description***   *Tell me more about [symptom]?*  *What does it feel like?*  *Could you put that in other words for me?*   - ***Severity***   *Describe how bad or severe the [symptom] is?*  *How severe would you rate it on a bad day, from 0-10?*  *How severe would you rate it on a good day, from 0-10?* |
| *(insert any new symptoms)* | □ | - ***Description***   *Tell me more about [symptom]?*  *What does it feel like?*  *Could you put that in other words for me?*   - ***Severity***   *Describe how bad or severe the [symptom] is?*  *How severe would you rate it on a bad day, from 0-10?*  *How severe would you rate it on a good day, from 0-10?* |
| *(insert any new symptoms)* | □ | - ***Description***   *Tell me more about [symptom]?*  *What does it feel like?*  *Could you put that in other words for me?*   - ***Severity***   *Describe how bad or severe the [symptom] is?*  *How severe would you rate it on a bad day, from 0-10?*  *How severe would you rate it on a good day, from 0-10?* |

1. **Do you experience any other symptoms of [patient term for axSpA] that we have not yet talked about?**
   - **IF YES:** *Tell me more about that.*
   - **IF NOT ALREADY DISCUSSED SPONTANEOUSLY:** *Do you ever experience [tiredness and fatigue/ sleep disturbance/ pain] as a result of your [patient term for axSpA]?*
2. **Tell me about the 3 symptom(s) due to [patient term for axSpA] that bother you the most. Why?**
   - *Of the three symptoms you discussed, which is the most bothersome and which is the least bothersome. Why is that? Tell me more.*
3. **Of all the symptoms you have mentioned today, which one would you most like to experience improvement in? Why?**
   - *What would that improvement look or feel like?*
   - *Why would that improvement be important to you?*

## Impacts of axSpA

- We are now going to talk further about what it is like to live with [patient term for axSpA] and how it may affect your life.

### Functional impacts

1. **How does [patient term for axSpA] affect your daily life, if at all?** [FOR EACH IMPACT EXPLORE THE FOLLOWING AS NEEDED:]
   - *Is there anything that you are unable to do or that you find difficult due to [patient term for axSpA]?*
     - *Why do you find this difficult or impossible to do?*
     - *Is this due to one or more symptoms of [patient term for axSpA]? Tell me more about that.*
     - *How often do you experience this? All of the time? Some of the time? Why?*
   - *Are there any things you avoid doing due to [patient term for axSpA]*
     - *Why do you avoid doing this?*
     - *Is this because of one or more symptoms of [patient term for axSpA?] How so?*
     - *Do you always have to avoid doing this, or some of the time? Why?*
2. **How does [patient term for axSpA] affect you physically, if at all?** [FOR EACH IMPACT EXPLORE THE FOLLOWING AS NEEDED:]
   - *Are any physical activities affected? How? Please tell me about this.*
   - *Are you able to do this activity less often, not as well or not at all?*
   - *Why is this? Is this due to one or more symptoms of [patient term for axSpA]?*
3. **Does [patient term for axSpA] affect your ability to work at all?** [FOR EACH IMPACT EXPLORE THE FOLLOWING AS NEEDED:]
   - *How has it affected your ability to work?*
   - *Are you less productive?*
   - *Have you had to take time off work?*
   - *Have you had to reduce the number of hours you work?*
   - *Have you had to change job or change roles within your job?*
   - *Are you unable to work or have had to take early retirement?*

- *Are there particular symptoms of [patient term for axSpA] which affect your work? Which ones? Why?*
- [IF NO LONGER WORKS] *When you have worked in the past/in your previous work, tell me about any ways [patient term for axSpA] impacted your work.*

### Emotional impacts

1. **Does [patient term for axSpA] affect your mood or how you feel emotionally in any way?** [FOR EACH IMPACT EXPLORE THE FOLLOWING AS NEEDED:]

- *Tell me about the emotions you have experienced because of [patient term for axSpA].*
- *Have you felt this way because of one or more symptoms of [patient term for axSpA]? Tell me about that*
- *Have the emotions you experienced changed over time at all?*
  - *e.g. has having [patient term for axSpA] affected you more or less at certain times? Tell me about that.*

### Cognitive impacts

1. **Does [patient term for axSpA] affect your ability to think at all?** [FOR EACH IMPACT EXPLORE THE FOLLOWING AS NEEDED:]
   - *How is your ability to think affected?*
   - *For example, has it affected: concentration, memory, speech? How often do you experience this?*
   - *What symptom or symptoms of [patient term for axSpA] causes this do you think?*

### Social impacts

1. **Does [patient term for axSpA] affect your social or leisure activities at all?** [FOR EACH IMPACT EXPLORE THE FOLLOWING AS NEEDED:]
   - *What type of activities have been affected? For example, seeing friends, cancelling social events?*
   - *How often are your social activities affected?*
   - *What symptom or symptoms of [patient term for axSpA] causes this do you think?*
2. **Does [patient term for axSpA] affect any other parts of your life that we haven’t yet discussed? Please tell me about these.**

IF THERE IS AT LEAST 5-MINUTES REMANING IN PART 2 OF THE INTERVIEW (35 MINUTES OR LESS INTO THE INTERVIEW), ASK TREATMENT OF AXSPA QUESTIONS. IF NOT, MOVE TO PART 3 OF THE INTERVIEW GUIDE AND RETURN TO THIS SECTION IF THERE IS TIME LEFT AT THE END.

## Targeted discussion about treatment of axSpA

1. **Tell me about the type of treatment you currently take for your axSpA.**

FOR EACH CURRENT TREATMENT ASK THE FOLLOWING PROBES:

- 1. *How do you take this treatment (pill or injection)?*
  2. *How often do you take it?*
  3. *Using a scale of 0-5 with 0 being very dissatisfied and 5 being very satisfied, overall how satisfied are you with your current treatment? Please explain why.*
  4. *Which symptoms does it completely relieve, if any?*
  5. *Are there any symptoms it improves but doesn’t completely relieve? Tell me about those.*
  6. *Are there any symptoms that it doesn’t improve at all? Tell me about those.*
- Before we move onto the next section of the interview, would you like to take a 10-15 minute break?

# Part 3: Cognitive debriefing of the PROMIS questionnaire (35-45 minutes)

- The aim of the second half of this interview is to your feedback on three questionnaires which ask about fatigue, sleep and pain. Can you confirm you have copies of the questionnaires in front of you, as well as the paper score thermometers?

**BEFORE STARTING THE COGNITIVE DEBRIEFING, PLEASE ENSURE THE PARTICIPANT HAS THE FOLLOWING DOCUMENTS IN FRONT OF THEM:**

- PROMIS Fatigue (Appendix A)
- PROMIS Sleep disturbance (Appendix B)
- PROMIS Pain interference (Appendix C)
- Visual aid for meaningful change questions (Appendix E)

**Note to interviewer: If patient does not have PROMIS measures with them, please ask the participant if it is possible to email to PROMIS questionnaires to them and continue with the interview. If not, rearrange the interview for a later date and end the interview. If they do have the questionnaires, ask the participant to read each instruction/question out loud and before asking the probes provided for each instruction/question in the table.**

- We will go through each question in turn. I want to understand if each instruction and question is clear and easy for you to understand, and if it is relevant to you and your experience of your back condition.
- I will ask you to read each instruction and question out loud as you complete the questionnaires. I would like you to tell me each answer you select and why you have chosen that answer.
- When answering questions, please respond in a way that best reflects your personal experiences and feel free to give your opinions. There are no right or wrong answers. We want your help to identify any problems with the questions and any things that could be made better – so don’t hesitate to tell us about anything that is unclear or difficult to understanding about the questions, or anything that is missing or should be added.

**IF YOU ARE RUNNING OUT OF TIME, PLEASE ENSURE YOU PRIORITIZE EXPLORING THE RELEVANCE** **OF ITEMS WITH THE PARTICIPANT. WE WOULD LIKE TO HAVE RESPONSES TO ALL OF THE ITEMS; IF YOU ARE RUNNING OUT OF TIME, PLEASE ASK THE PARTICIPANT TO COMPLETE ALL OF THE REMAINING QUESTIONS OUT LOUD AND PROBE ANY ITEMS WHERE THE RESPONSE IS ‘NOT AT ALL’, ‘NEVER’, ’VERY GOOD’.**

## PROMIS Fatigue

- Please take a look at the ‘Fatigue-Short Form’ questionnaire. Please read the instructions out loud. After this, please read each of the questions out loud, tell me your answer and mark down it down on the questionnaire.

(ASK THE PARTICIPANT TO READ EACH INSTRUCTION/QUESTION OUT LOUD BEFORE ASKING THE PROBES PROVIDED FOR EACH INSTRUCTION/QUESTION IN THE TABLE. NOTE DOWN THE PARTICIPANT’S ANSWER FOR EACH QUESTION SO THAT A TOTAL SCORE CAN BE CALCULATED. ALSO ENSURE THE PARTICIPANT IS MARKING DOWN THEIR ANSWERS AS THEY GO ALONG).

| **Instruction/item** | **Understanding** | **Relevance** | **Response options** | **Recall period** |
| --- | --- | --- | --- | --- |
| **INSTRUCTIONS:** Please respond to each question by marking one box per row. | *Is there anything unclear about the way this instruction is worded? Please explain.* |  |  |  |
| **ITEM STEM:** In the past 7 days… |  |  |  |  |
| **HI7**: I feel fatigued  Not at all / A little bit / Somewhat / Quite a bit / Very much | *Is there anything unclear about the way this question is worded? Please explain.* | *Do you ever feel fatigued because of your [patient term for axSpA]?*  *How fatigued do you feel?* | *What did you choose and why?*  *You chose XXX – what would have to happen for you to choose XXX (chose a response option one level up or down)* | *What time period were you thinking about when answering this question?* |
| **AN2:** I feel tired  Not at all / A little bit / Somewhat / Quite a bit / Very much | *Is there anything unclear about the way this question is worded? Please explain.* | *To what extent do you ever feel tired because of your [patient term for axSpA]?* | *What did you choose and why?*  *Do the answers make sense to you?* |  |
| **AN3:** I have trouble starting things because I am tired  Not at all / A little bit / Somewhat / Quite a bit / Very much | *Is there anything unclear about the way this question is worded? Please explain.* | *Do you ever have trouble starting things because you are tired as a result of your [patient term for axSpA]?*  *How much of a problem is this in your life?* | *What did you choose and why?*  *What is the difference between ‘a little bit’’ and ‘somewhat’ trouble starting things because you are tired?* |  |
| **AN4:** I have trouble finishing things because I am tired  Not at all / A little bit / Somewhat / Quite a bit / Very much | *Is there anything unclear about the way this question is worded? Please explain.* | *Do you ever have trouble finishing things because of tiredness as a result of your [patient term for axSpA]?*  *How often does this occur?* | *What did you choose and why?* |  |
| **AN5:** I have energy  Not at all / A little bit / Somewhat / Quite a bit / Very much | *Is there anything unclear about the way this question is worded? Please explain.* | *To what extent do you ever feel like you do not have energy because of your [patient term for axSpA]?* | *What did you choose and why?* |  |
| **AN7:** I am able to do my usual activities  Not at all / A little bit / Somewhat / Quite a bit / Very much | *Is there anything unclear about the way this question is worded? Please explain.* | *Are you able to undertake your usual activities?*  *To what extent does your [patient term for axSpA] impact your ability to under your usual activities?* | *What did you choose and why?* |  |
| **AN8:** I need to sleep during the day  Not at all / A little bit / Somewhat / Quite a bit / Very much | *Is there anything unclear about the way this question is worded? Please explain.* | *Do you ever need sleep during the day because of your [patient term for axSpA]?*  *How often does this occur?*  *How long do you need to sleep for?* | *What did you choose and why?* |  |
| **AN14:** I need help doing my usual activities  Not at all / A little bit / Somewhat / Quite a bit / Very much | *Is there anything unclear about the way this question is worded? Please explain.* | *To what extent do you ever need help doing your usual activities because of your [patient term for axSpA]?* | *What did you choose and why?*  *What is the difference between ‘a little bit’’ and ‘somewhat’ with you needing help doing your usual activities?* |  |
| **AN15:** I am frustrated by being too tired to do the things I want to do  Not at all / A little bit / Somewhat / Quite a bit / Very much | *Is there anything unclear about the way this question is worded? Please explain.* | *Do you ever get frustrated by being too tired to do the things you want to do?*  *How often does this happen?* | *What did you choose and why?* |  |
| **AN16:** I have to limit my social activity because I am tired  Not at all / A little bit / Somewhat / Quite a bit / Very much | *Is there anything unclear about the way this question is worded? Please explain.* | *To what extent do you to have to limit your social activity because you are tired?* | *What did you choose and why?* | *Was it easy or difficult to remember your experience of fatigue over the past 7-days?* |

- I would now like to ask you some general questions about the Fatigue-Short Form questionnaire. While you were answering and discussing those questions I noted down your responses and I am just going to calculate your score now. The answers to each question in the questionnaire is given a score from 1 to 5. To calculate a total score, each of the responses are summed together to get a final score that ranges from 10 to 50 (where higher scores mean worse tiredness and fatigue). On this questionnaire, your total score was [INSERT PARTICIPANT SCORE]. Please look at the document you were given titled ‘Visual aid for meaningful change questions’ (Appendix E) and look at ‘Figure 1. PROMIS fatigue visual aid’. This thermometer shows how the scores range for this questionnaire. Would you like to mark your total score on the thermometer that you received with the questionnaires?

1. **If you are starting at [participant’s total score], where on the thermometer would you want to get to for you to consider the change important and worth taking a treatment for? Please mark that down.**
   1. *Why would that change be important to you?*
   2. *How would this affect the way you feel?*
   3. *Would this change your day to day life? How?*
2. **Is there anything missing from the questionnaire that you think would be important to [patient term for axSpA] patients?**
3. **Are there any questions which you think should be removed? Maybe questions that don’t apply to you?**
4. **How easy or difficult do you find it to answer the questions? Tell me more about that.**
   1. *Is there anything that could be done to make this easier?*

## PROMIS Sleep Disturbance

- Please now take a look at the ‘Sleep Disturbance’ questionnaire. Please read the instructions out loud. After this, please reach each of the questions out loud, tell me your answer and mark down it down on the questionnaire.

(ASK THE PARTICIPANT TO READ EACH INSTRUCTION/QUESTION OUT LOUD BEFORE ASKING THE PROBES PROVIDED FOR EACH INSTRUCTION/QUESTION IN THE TABLE. NOTE DOWN THE PARTICIPANT’S ANSWER FOR EACH QUESTION SO THAT A TOTAL SCORE CAN BE CALCULATED. ALSO ENSURE THE PARTICIPANT IS MARKING DOWN THEIR ANSWERS AS THEY GO ALONG).

| **Instruction/item** | **Understanding** | **Relevance** | **Response options** | **Recall period** |
| --- | --- | --- | --- | --- |
| **INSTRUCTIONS:** Please respond to each question by marking one box per row. | *Is there anything unclear about the way this instruction is worded? Please explain.* |  |  |  |
| **ITEM STEM:** In the past 7 days… |  |  |  |  |
| **Sleep108**: My sleep was restless  Not at all / A little bit / Somewhat / Quite a bit / Very much | *Is there anything unclear about the way this question is worded? Please explain.* | *Do you ever experience restless sleep because of your [patient term for axSpA]?*  *How often do you experience this?* | *What did you choose and why?*  *You chose XXX – what would have to happen for you to choose XXX (chose a response option one level up or down)* |  |
| **Sleep115**: I was satisfied with my sleep  Not at all / A little bit / Somewhat / Quite a bit / Very much | *Is there anything unclear about the way this question is worded? Please explain.* | *Can you tell me about your satisfaction with your sleep?* | *What did you choose and why?*  *Do the answers make sense to you?* | *What time period were you thinking about when answering this question?* |
| **Sleep44:** I had difficulty falling asleep  Not at all / A little bit / Somewhat / Quite a bit / Very much | *Is there anything unclear about the way this question is worded? Please explain.* | *To what extent do you have difficulties falling asleep because of your [patient term for axSpA]?* | *What did you choose and why?*  *What is the difference between ‘a little bit’ and ‘somewhat’ difficulty falling asleep?* |  |
| **Sleep71:** I had trouble getting into a comfortable position to sleep  Not at all / A little bit / Somewhat / Quite a bit / Very much | *Is there anything unclear about the way this question is worded? Please explain.* | *How often do you have trouble getting into a comfortable position to sleep because of your [patient term for axSpA]?* | *What did you choose and why?* |  |
| **Sleep110:** I got enough sleep  Never / Rarely / Sometimes / Often / Always | *Is there anything unclear about the way this question is worded? Please explain.* | *How often do you feel that you do not get enough sleep because of your [patient term for axSpA]?* | *What did you choose and why?*  *You chose XXX – what would have to happen for you to choose XXX (chose a response option one level up or down)* |  |
| **Sleep92:** I woke up and had trouble falling back to sleep  Never / Rarely / Sometimes / Often / Always | *Is there anything unclear about the way this question is worded? Please explain.* | *Do you ever wake up during the night and then have trouble getting back to sleep because of your [patient term for axSpA]?*  *How often does this happen?* | *What did you choose and why?*  *Do the answers make sense to you?* |  |
| **Sleep109:** My sleep quality was  Very poor / Poor / Fair / Good / Very good | *Is there anything unclear about the way this question is worded? Please explain.* | *Does your [patient term for axSpA] ever affect your sleep quality?*  *How often does this happen?* | *What did you choose and why?*  *What is the difference between ‘poor’ and ‘fair’ sleep quality?* | *Was it easy or difficult to remember your experience of sleep quality over the past 7-days?* |

- I would now like to ask you some general questions about the Sleep Disturbance questionnaire. While you were answering and discussing those questions I noted down your responses and I am just going to calculate your score now. The answers to each question in the questionnaire is given a score from 1 to 5. To calculate a total score, each of the responses are summed together to get a final score that ranges from 7 to 35 (where higher scores mean worse sleep disturbance). On this questionnaire, your total score was [INSERT PARTICIPANT SCORE]. Please look at the document you were given titled ‘Visual aid for meaningful change questions’ (Appendix E) and look at ‘Figure 2. Sleep disturbance’. This thermometer shows how the scores range for this questionnaire. Would you like to mark your total score on the thermometer that you received with the questionnaires?

1. **If you are starting at [participant’s total score], where on the thermometer would you want to get to for you to consider the change important and worth taking a treatment for? Please mark that down.**
   1. *Why would that change be important to you?*
   2. *How would this affect the way you feel?*
   3. *Would this change your day to day life? How?*
2. **Is there anything missing from the questionnaire that you think would be important to [patient term for axSpA] patients?**
3. **Are there any questions which you think should be removed? Maybe questions that don’t apply to you?**
4. **How easy or difficult do you find it to answer the questions? Tell me more about that.**
   1. *Is there anything that could be done to make this easier?*

## PROMIS Pain Interference

- Please now take a look at the ‘Pain Interference’ questionnaire. Please read the instructions out loud. After this, please reach each of the questions out loud, tell me your answer and mark down it down on the questionnaire.

(ASK THE PARTICIPANT TO READ EACH INSTRUCTION/QUESTION OUT LOUD BEFORE ASKING THE PROBES PROVIDED FOR EACH INSTRUCTION/QUESTION IN THE TABLE. NOTE DOWN THE PARTICIPANT’S ANSWER FOR EACH QUESTION SO THAT A TOTAL SCORE CAN BE CALCULATED. ALSO ENSURE THE PARTICIPANT IS MARKING DOWN THEIR ANSWERS AS THEY GO ALONG).

| **Instruction/item** | **Understanding** | **Relevance** | **Response options** | **Recall period** |
| --- | --- | --- | --- | --- |
| **INSTRUCTIONS:** Please respond to each question by marking one box per row. | *Is there anything unclear about the way this instruction is worded? Please explain.* |  |  |  |
| **ITEM STEM:** In the past 7 days… |  |  |  |  |
| **PAININ3**: How much did pain interfere with your enjoyment of life?  Not at all / A little bit / Somewhat / Quite a bit / Very much | *Is there anything unclear about the way this question is worded? Please explain.* | *Has pain from your [patient term for axSpA] ever interfered with your enjoyment of life?*  *How often has this happened?* | *What did you choose and why?*  *You chose XXX – what would have to happen for you to choose XXX (chose a response option one level up or down)* |  |
| **PAININ31:** How much did pain interfere with your ability to participate in social activities?  Not at all / A little bit / Somewhat / Quite a bit / Very much | *Is there anything unclear about the way this question is worded? Please explain.* | *What type of social activities has pain interfered with, if ever?*  *How often did it interfere?* | *What did you choose and why?*  *Do the answers make sense to you?* |  |
| **PAININ9:** How much did pain interfere with your day-to-day activities?  Not at all / A little bit / Somewhat / Quite a bit / Very much | *Is there anything unclear about the way this question is worded? Please explain.* | *What type of day to day activities has pain interfered with, if ever?*  *How often did it interfere?* | *What did you choose and why?*  *What type of interference would you experience to choose ‘XXX’ [a response one level higher/lower]* |  |
| **PAININ20:** How much did pain feel like a burden to you?  Not at all / A little bit / Somewhat / Quite a bit / Very much | *Is there anything unclear about the way this question is worded? Please explain.* | *How often has pain been a burden upon you?*  *Can you tell me about times where the burden of pain has impacted your daily life?* | *What did you choose and why?* | *What time period were you thinking about when answering this question?* |
| **PAININ56:** How irritable did you feel because of pain?  Not at all / A little bit / Somewhat / Quite a bit / Very much | *Is there anything unclear about the way this question is worded? Please explain.* | *Can you tell me about any times where you felt irritable because of pain?* | *What did you choose and why?*  *What is the difference between ‘a little bit’ and ‘somewhat’ irritable?* |  |
| **PAININ17:** How much did pain interfere with your relationship with other people?  Not at all / A little bit / Somewhat / Quite a bit / Very much | *Is there anything unclear about the way this question is worded? Please explain.* | *Can you tell me about any times where pain has interfered with your relationship with other people?*  *How often does this occur?* | *What did you choose and why?*  *What would you select if pain substantially changed your relationship with one person on one occasion, but had not interfered with any other relationships since?* |  |
| **PAININ35:** How much did pain interfere with your ability to make trips from home that kept you gone for more than 2 hours?  Not at all / A little bit / Somewhat / Quite a bit / Very much | *Is there anything unclear about the way this question is worded? Please explain.* | *Can you tell me about times where pain has impacted your ability to make trips away from home, where you would be gone for more than 2-hours?*  *How often does this occur?* | *What did you choose and why?*  *What would be the difference in interference if you selected ‘somewhat’ and ‘quite a bit’* |  |
| **PAININ29:** How often was your pain so severe you could think of nothing else?  Never / Rarely / Sometimes / Often / Always | *Is there anything unclear about the way this question is worded? Please explain.* | *Can you tell me about times where your pain is so severe that you could think of nothing else?*  *How often does this happen?* | *What did you choose and why?*  *What is the difference between rarely and sometimes?* |  |
| **PAININ50:** How often did pain prevent you from sitting for more than 30-minutes?  Never / Rarely / Sometimes / Often / Always | *Is there anything unclear about the way this question is worded? Please explain.* | *Has pain ever impacted your ability to sit for more than 30-minutes? Tell me more about this.* | *What did you choose and why?*  *Do the answers make sense to you?* |  |
| **PAININ47:** How often did pain prevent you from standing for more than 30-minutes?  Never / Rarely / Sometimes / Often / Always | *Is there anything unclear about the way this question is worded? Please explain.* | *Do you experience pain that prevents you from standing for more than 30-minutes? Tell me about that.*  *How often does this happen?* | *What did you choose and why?*  *You chose XXX. What would have to happen for you to choose XXX [one level up or down]* |  |
| **PAININ47:** How often did pain keep you from getting into a standing position?  Never / Once a week or less / Once every few days / Once a day / Every few hours | *Is there anything unclear about the way this question is worded? Please explain.* | *Has pain ever impacted your ability from getting into a standing position? Tell me about that.* | *What did you choose and why?*  *If you experienced this several times in one day only, during a seven day period, what answer would you select?* |  |

- I would now like to ask you some general questions about the Pain Interference questionnaire. While you were answering and discussing those questions I noted down your responses and I am just going to calculate your score now. The answers to each question in the questionnaire is given a score from 1 to 5. To calculate a total score, each of the responses are summed together to get a final score that ranges from 11 to 55 (where higher scores mean worse pain interference). On this questionnaire, your total score was [INSERT PARTICIPANT SCORE]. Please look at the document you were given titled ‘Visual aid for meaningful change questions’ (Appendix E) and look at ‘Figure 3. Pain interference'. This thermometer shows how the scores range for this questionnaire. Would you like to mark that on the thermometer that you received with the questionnaires?
  - - 1. **If you are starting at [participant’s total score], where on the thermometer would you want to get to for you to consider the change important and worth taking a treatment for? Please mark that down.**
  1. *Why would that change be important to you?*
  2. *How would this affect the way you feel?*
  3. *Would this change your day to day life? How?*

1. **Is there anything missing from the questionnaire that you think would be important to [patient term for axSpA] patients?**
2. **Are there any questions which you think should be removed? Maybe questions that don’t apply to you?**
3. **How easy or difficult do you find it to answer the questions? Tell me more about that.**
   - *Is there anything that could be done to make this easier?*

**End of interview**

*Thank the participant for taking part in the interview and answer any questions they may have.*

**Round two interview guide**

# Part 1: Interview introduction (5 minutes)

## Objective

Introduce yourself to the participant, review the purpose of the study and confidentiality arrangements, ask for the participant’s permission to audio record the interview and record your name, the participant’s ID, the date, and the local time and location for you and the participant.

## Interviewer instructions

1. **Thank the participant for agreeing to take part in this interview.**
2. **Introduce yourself as working on behalf of Adelphi Values:**

- My name is [your name] and I am working on behalf of a health research company called Adelphi Values that works closely with pharmaceutical companies to assess the impact of health conditions and treatments on peoples’ lives.

1. **Screening for COVID-19 (ideally, if the participant is not feeling well, he or she should call the interviewer (or recruiter or site?) to reschedule prior to the day of the scheduled interview)**

- Before we start, I need to ask you some questions about COVID-19. I want to make sure that you are feeling well today and able to spend the next 90 minutes with me to help us with our study. [PLEASE NOTE DOWN THE PARTICIPANT’S RESPONSES TO THE FOLLOWING QUESTIONS]

1. **Have you had COVID-19 (either suspected, probable or confirmed)? [Yes or No]**
   1. **If Yes, select the option that best describes you:**

- **Suspected/Probable: I have recently had COVID-19-like symptoms (for example, shortness of breath, cough, fever, extreme fatigue)**
- **Confirmed: I have or have had a confirmed diagnosis of COVID-19**

1. **If answer to 1 is Yes – Have you been symptom-free for the past 14 days? [Yes or No]**
   1. **If answer is No – Schedule the interview to 3 weeks’ time at the earliest**
   2. **If answer is Yes – When did you start experiencing symptoms OR receive your diagnosis of COVID-19? [DD-MMM-YYYY]**
2. **If answer to 1 is No – Are you feeling ill or unwell today? If No – continue with the interview, If Yes – reschedule interview.**
3. **Explain the aim of the interview:**

- The aim of the first part of the interview is to understand your experience of living with your axial spondyloarthritis, including the symptoms and the impact that this has on your life.
- The second part of the interview is designed to get your feedback on three questionnaires which ask about fatigue, sleep and pain. Can you confirm you have copies of the three questionnaires in front of you? You should also have three pieces of paper, each with a large thermometer printed on them; can you confirm you also have these in front of you?

1. **Reassure the participant of confidentiality and anonymity:**

- Your name and contact information will remain with the researchers at Adelphi Values and will only be accessible to staff directly involved with this project.
- Any information you provide will be reported in a way that protects your privacy by avoiding any mention of your name or other information that could identify you.
- The recording of the interview will be typed up, word for word, and will be shared with the sponsor of this project but your name or any information that identifies you will be removed first.

1. **Discuss the interview:**

- The interview will up to 90-minutes.
- The interview will be audio-recorded to allow me to pay careful attention to what you say and to make certain we accurately record the things that you tell me during the interview.
- Please try and speak relatively loudly and clearly so that your comments can be heard and are clear on the recording.
- We want to let you know that the questions during the interview may be repetitive, but that this is necessary, as we want your opinion on the questions and answers.
- You do not have to answer any questions you do not feel comfortable answering.
- You can ask questions at any point in the interview.
- There are no right or wrong answers; we are interested in your personal opinion.
- You can pause or leave the interview at any point.
- You will be compensated after completing the interview.

1. **Turn on audio-recording device *
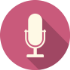
***

- The recording has now begun.
- This is [NAME OF INTERVIEWER] with participant [PARTICIPANT ID NUMBER] on [DATE OF INTERVIEW] at [TIME OF INTERVIEW AND TIME ZONE]. The interview is being conducted by [TELEPHONE/VIDEO]. I am taking the call in [STATE LOCATION OF INTERVIEWER, INCLUDING CITY/STATE AND COUNTRY] and the participant is taking the call in [STATE LOCATION OF PARTICIPANT].
- Do you agree to participate in the interview?
- Do you agree to have this interview audio-recorded?
  - [IF PARTICIPANT DOES NOT AGREE TO HAVE THE INTERVIEW RECORDED: Thank the participant for taking part and end the interview and switch off the recorder.]
- Are you in a location where it is safe to participate in the interview?
- Do you expect to be distracted or have your attention required elsewhere in the next 75-90 minutes?
  - [IF NOT FOR EITHER OF THE ABOVE: Arrange to reschedule the interview and switch off the recorder.]
- Do you have any questions before we start?
- OK, I’ll start now, ok?

# Part 2: Concept elicitation of patient experience of axSpA (20 minutes)

## Objective

The objective of this section of the interview is to encourage the participant to talk spontaneously about the experience of axSpA, including the symptom and treatment experience and how the condition impacts daily life. More probed questions are provided if the participant does not spontaneously discuss all topics of interest. Probes to explore each reported symptom in detail are provided in .

- Today I would like to talk to you about your [axial spondyloarthritis/ ankylosing spondylitis] . When talking about your [axial spondyloarthritis/ ankylosing spondylitis] to your friends and family, what do you call it? What do you refer it as?

TERM(S) USED: ______________________________________

- Ok, so for the rest of the interview, I will refer to your [patient term for axSpA] if that’s ok?

## Symptoms of axSpA

- We are now going to talk about your symptoms and experiences of [patient term for axSpA**].**

1. **Thinking back to when you first started experiencing symptoms associated with your [**patient term for axSpA**], what symptoms did you experience?**
   - **What led you to seek advice from your doctor?*
2. *****Please tell me about your day to day experience of having [**patient term for axSpA**].**
   - *What is a good day like with [patient term for axSpA]*
   - *What is a bad day like with [patient term for axSpA]*
     - *How often do you have bad days?*

| **TICK EACH SYMPTOM THAT THE PATIENT MENTIONS SPONTANEOUSLY TO QUESTIONS 1 AND 2 IN THE TRACKING TABLE (Table 1). INSERT ANY ADDITIONAL SYMPTOMS THAT ARE DISCUSSED INTO THE TABLE. ENSURE THE KEY ASPECTS ARE DISCUSSED FOR EACH PROBE BY TICKING THE TOPIC IN THE PROBE COLUMN. IF THE PARTICIPANT DOESN’T SPONTANEOUSLY DISCUSS A KEY TOPIC FOR THAT SYMPTOM (E.G. SEVERITY) THEN PLEASE USE THE SPECIFIED PROBES TO ASK ABOUT THIS.** | | |
| --- | --- | --- |
| Table 1. Symptom tracking table | | |
| Symptom | Tick when mentioned by patient | Symptom-specific probe   - Please tick when information obtained |
| Tiredness and fatigue | □ | - ***Description***   *What does this feel like?*   - ***Severity***   *Describe how bad or severe the tiredness and fatigue is.*  *How severe would you rate it on a bad day, from 0-10? (with 0 meaning no tiredness/fatigue at all and 10 meaning the worst tiredness/fatigue imaginable)*  *How severe would you rate it on a good day, from 0-10? (with 0 meaning no tiredness/fatigue at all and 10 meaning the worst tiredness/fatigue imaginable)*   - ***Frequency***   *How often do you experience tiredness and fatigue with your [patient term for axSpA]?*  *How long does tiredness and fatigue last?* |
| Sleeping problems | □ | - ***Description***   *Tell me more about your sleeping problems.*  *Do you find it hard to go to sleep?*  *Do you have difficulties staying asleep?*  *Do you have difficulties waking up?*   - ***Severity***   *Describe how bad or severe your sleeping problems are.*  *How severe would you rate it on a bad day, from 0-10? (0 meaning no sleeping problems at all and 10 meaning the worst sleeping problems imaginable)*  *How severe would you rate it on a good day, from 0-10? (0 meaning no sleeping problems at all and 10 meaning the worst sleeping problems imaginable)*   - ***Frequency***   *How often do you experience sleeping problems because of your [patient term for axSpA]?* |
| Pain | □ | - ***Description***   *What does it feel like?*   - ***Location***   *Where do you experience that on your body?*   - ***Severity***   *Describe how bad or severe the pain is*  *How severe would you rate it on a bad day, from 0-10? (0 meaning no pain at all and 10 meaning the worst pain imaginable)*  *How severe would you rate it on a good day, from 0-10? (0 meaning no pain at all and 10 meaning the worst pain imaginable)*   - ***Frequency***   *How often do you experience pain due to your [patient term for axSpA]?*  *How long does the pain you experience last?* |
| *(insert any new symptoms)* | □ | - ***Description***   *Tell me more about [symptom]?*  *What does it feel like?*  *Could you put that in other words for me?*   - ***Severity***   *Describe how bad or severe the [symptom] is?*  *How severe would you rate it on a bad day, from 0-10? (0 meaning no experience of the symptom at all and 10 meaning the worst experience of the symptom imaginable)*  *How severe would you rate it on a good day, from 0-10? (0 meaning no experience of the symptom at all and 10 meaning the worst experience of the symptom imaginable)* |
| *(insert any new symptoms)* | □ | - ***Description***   *Tell me more about [symptom]?*  *What does it feel like?*  *Could you put that in other words for me?*   - ***Severity***   *Describe how bad or severe the [symptom] is?*  *How severe would you rate it on a bad day, from 0-10? (0 meaning no experience of the symptom at all and 10 meaning the worst experience of the symptom imaginable)*  *How severe would you rate it on a good day, from 0-10? (0 meaning no experience of the symptom at all and 10 meaning the worst experience of the symptom imaginable)* |
| *(insert any new symptoms)* | □ | - ***Description***   *Tell me more about [symptom]?*  *What does it feel like?*  *Could you put that in other words for me?*   - ***Severity***   *Describe how bad or severe the [symptom] is?*  *How severe would you rate it on a bad day, from 0-10? (0 meaning no experience of the symptom at all and 10 meaning the worst experience of the symptom imaginable)*  *How severe would you rate it on a good day, from 0-10? (0 meaning no experience of the symptom at all and 10 meaning the worst experience of the symptom imaginable)* |

1. **Do you experience any other symptoms of [patient term for axSpA] that we have not yet talked about?**
   - **IF YES:** *Tell me more about that.*
   - **IF NOT ALREADY DISCUSSED SPONTANEOUSLY:** *Do you ever experience [tiredness and fatigue/ sleep disturbance/ pain] as a result of your [patient term for axSpA]?*
2. **Tell me about the 3 symptom(s) due to [patient term for axSpA] that bother you the most. Why?**
   - *Of the three symptoms you discussed, which is the most bothersome and which is the least bothersome. Why is that? Tell me more.*
3. **Of all the symptoms you have mentioned today, which one would you most like to experience improvement in? Why?**
   - *What would that improvement look or feel like?*
   - *Why would that improvement be important to you?*

## Impacts of axSpA

- We are now going to talk further about what it is like to live with [patient term for axSpA] and how it may affect your life.

### Functional impacts

1. **How does [patient term for axSpA] affect your daily life, if at all?** [FOR EACH IMPACT EXPLORE THE FOLLOWING AS NEEDED:]
   - **Is there anything that you are unable to do or that you find difficult due to [patient term for axSpA]?*
     - ***Is this due to one or more symptoms of [patient term for axSpA]? Tell me more about that.***
     - **How often do you experience this? All of the time? Some of the time? Why?*
   - **Are there any things you avoid doing due to [patient term for axSpA]*
     - **Why do you avoid doing this?*
     - ***Is this because of one or more symptoms of [patient term for axSpA?] How so?***
     - **Do you always have to avoid doing this, or some of the time? Why?*
2. **How does [patient term for axSpA] affect you physically, if at all?** [FOR EACH IMPACT EXPLORE THE FOLLOWING AS NEEDED:]
   - ***Why is this? Is this due to one or more symptoms of [patient term for axSpA]?***
3. **Does [patient term for axSpA] affect your ability to work at all?** [FOR EACH IMPACT EXPLORE THE FOLLOWING AS NEEDED:]

- ***Why is this? Are there particular symptoms of [patient term for axSpA] which affect your work? Which ones? Why?***
- *[IF NO LONGER WORKS] *When you have worked in the past/in your previous work, tell me about any ways [patient term for axSpA] impacted your work.*

### Emotional impacts

1. **Does [patient term for axSpA] affect your mood or how you feel emotionally in any way?** [FOR EACH IMPACT EXPLORE THE FOLLOWING AS NEEDED:]

- ***Why is this? Have you felt this way because of one or more symptoms of [patient term for axSpA]? Tell me about that***.

### Cognitive impacts

1. **Does [patient term for axSpA] affect your ability to think at all?** [FOR EACH IMPACT EXPLORE THE FOLLOWING AS NEEDED:]
   - **How is your ability to think affected?*
   - **For example, has it affected: concentration, memory, speech? How often do you experience this?*
   - ***What symptom or symptoms of [patient term for axSpA] causes this do you think?***

### Social impacts

1. ***Does [patient term for axSpA] affect your social or leisure activities at all?** [FOR EACH IMPACT EXPLORE THE FOLLOWING AS NEEDED:]
   - **What type of activities have been affected? For example, seeing friends, cancelling social events?*
   - **How often are your social activities affected?*
   - *What symptom or symptoms of [patient term for axSpA] causes this do you think?*
2. **Does [patient term for axSpA] affect any other parts of your life that we haven’t yet discussed? Please tell me about these.**

IF THERE IS AT LEAST 5-MINUTES REMANING IN PART 2 OF THE INTERVIEW (25 MINUTES OR LESS INTO THE INTERVIEW), ASK TREATMENT OF AXSPA QUESTIONS. IF NOT, MOVE TO PART 3 OF THE INTERVIEW GUIDE AND RETURN TO THIS SECTION IF THERE IS TIME LEFT AT THE END.

## Targeted discussion about treatment of axSpA

1. ***Tell me about the type of treatment you currently take for your axSpA.**

FOR EACH CURRENT TREATMENT ASK THE FOLLOWING PROBES:

- 1. *How do you take this treatment (pill or injection)?*
  2. *How often do you take it?*
  3. *Using a scale of 0-5 with 0 being very dissatisfied and 5 being very satisfied, overall how satisfied are you with your current treatment? Please explain why.*
  4. *Which symptoms does it completely relieve, if any?*
  5. *Are there any symptoms it improves but doesn’t completely relieve? Tell me about those.*
  6. *Are there any symptoms that it doesn’t improve at all? Tell me about those.*
- Before we move onto the next section of the interview, would you like to take a 10-15 minute break?

# Part 3: Cognitive debriefing of the PROMIS questionnaire (50-65 minutes)

- The aim of the second half of this interview is to receive your feedback on three questionnaires which ask about fatigue, sleep and pain. Can you confirm you have copies of the questionnaires in front of you, as well as the paper score thermometers?

**BEFORE STARTING THE COGNITIVE DEBRIEFING, PLEASE ENSURE THE PARTICIPANT HAS THE FOLLOWING DOCUMENTS IN FRONT OF THEM:**

- PROMIS Pain interference (Appendix A)
- PROMIS Fatigue (Appendix B)
- PROMIS Sleep disturbance (Appendix C)
- Visual aid for meaningful change questions (Appendix E)

**Note to interviewer: If patient does not have PROMIS measures with them, please ask the participant if it is possible to email to PROMIS questionnaires to them and continue with the interview. If not, rearrange the interview for a later date and end the interview. If they do have the questionnaires, ask the participant to read each instruction/question out loud and before asking the probes provided for each instruction/question in the table.**

- We will go through each question in turn. I want to understand if each instruction and question is clear and easy for you to understand, and if it is relevant to you and your experience of your back condition.
- I will ask you to read each instruction and question out loud as you complete the questionnaires. I would like you to tell me each answer you select and why you have chosen that answer.
- When answering questions, please respond in a way that best reflects your personal experiences and feel free to give your opinions. There are no right or wrong answers. We want your help to identify any problems with the questions and any things that could be made better – so don’t hesitate to tell us about anything that is unclear or difficult to understanding about the questions, or anything that is missing or should be added.

**IF YOU ARE RUNNING OUT OF TIME, PLEASE ENSURE YOU PRIORITIZE EXPLORING THE RELEVANCE** **OF ITEMS WITH THE PARTICIPANT. WE WOULD LIKE TO HAVE RESPONSES TO ALL OF THE ITEMS; IF YOU ARE RUNNING OUT OF TIME, PLEASE ASK THE PARTICIPANT TO COMPLETE ALL OF THE REMAINING QUESTIONS OUT LOUD AND PROBE ANY ITEMS WHERE THE RESPONSE IS ‘NOT AT ALL’, ‘NEVER’, ’VERY GOOD’.**

## PROMIS Pain Interference

- Please now take a look at the ‘Pain Interference’ questionnaire. Please read the instructions out loud. After this, please reach each of the questions out loud, tell me your answer and mark down it down on the questionnaire.

(ASK THE PARTICIPANT TO READ EACH INSTRUCTION/QUESTION OUT LOUD BEFORE ASKING THE PROBES PROVIDED FOR EACH INSTRUCTION/QUESTION IN THE TABLE. NOTE DOWN THE PARTICIPANT’S ANSWER FOR EACH QUESTION SO THAT A TOTAL SCORE CAN BE CALCULATED. ALSO ENSURE THE PARTICIPANT IS MARKING DOWN THEIR ANSWERS AS THEY GO ALONG).

| **Instruction/item** | **Understanding** | **Relevance** | **Response options** | **Recall period** |
| --- | --- | --- | --- | --- |
| **INSTRUCTIONS:** Please respond to each question by marking one box per row. | *Is there anything unclear about the way this instruction is worded? Please explain.* |  |  |  |
| **ITEM STEM:** In the past 7 days… |  |  |  |  |
| **PAININ3**: How much did pain interfere with your enjoyment of life?  Not at all / A little bit / Somewhat / Quite a bit / Very much | *Is there anything unclear about the way this question is worded? Please explain.* | *Has pain from your [patient term for axSpA] ever interfered with your enjoyment of life?*  *How often has this happened?* | *What did you choose and why?*  *You chose XXX – what would have to happen for you to choose XXX (chose a response option one level up or down)* |  |
| **PAININ31:** How much did pain interfere with your ability to participate in social activities?  Not at all / A little bit / Somewhat / Quite a bit / Very much | *Is there anything unclear about the way this question is worded? Please explain.* | *What type of social activities has pain interfered with, if ever?*  *How often did it interfere?* | *What did you choose and why?*  *Do the answers make sense to you?* |  |
| **PAININ9:** How much did pain interfere with your day-to-day activities?  Not at all / A little bit / Somewhat / Quite a bit / Very much | *Is there anything unclear about the way this question is worded? Please explain.* | *What type of day to day activities has pain interfered with, if ever?*  *How often did it interfere?* | *What did you choose and why?*  *What type of interference would you experience to choose ‘XXX’ [a response one level higher/lower]* |  |
| **PAININ20:** How much did pain feel like a burden to you?  Not at all / A little bit / Somewhat / Quite a bit / Very much | *Is there anything unclear about the way this question is worded? Please explain.* | *How often has pain been a burden upon you?*  *Can you tell me about times where the burden of pain has impacted your daily life?* | *What did you choose and why?* | *What time-period were you thinking about when answering this question?*  *Do you think the timeframe of ‘in the past 7 days’ is appropriate for this question?*  *if your pain was less of a burden at the start of the 7 days, but more of a burden in the past 2 days, which response option would you choose and why?* |
| **PAININ56:** How irritable did you feel because of pain?  Not at all / A little bit / Somewhat / Quite a bit / Very much | *Is there anything unclear about the way this question is worded? Please explain.* | *Can you tell me about any times where you felt irritable because of pain?*  *How easy is it to know if you are feeling irritable due to pain, or for other reasons?* | *What did you choose and why?*  *What is the difference between ‘a little bit’ and ‘somewhat’ irritable?* |  |
| **PAININ17:** How much did pain interfere with your relationship with other people?  Not at all / A little bit / Somewhat / Quite a bit / Very much | *Is there anything unclear about the way this question is worded? Please explain.* | *Can you tell me about any times where pain has interfered with your relationship with other people?*  *How often does this occur?* | *What did you choose and why?*  *What would you select if pain substantially changed your relationship with one person on one occasion, but had not interfered with any other relationships since?* |  |
| **PAININ35:** How much did pain interfere with your ability to make trips from home that kept you gone for more than 2 hours?  Not at all / A little bit / Somewhat / Quite a bit / Very much | *Is there anything unclear about the way this question is worded? Please explain.* | *Can you tell me about times where pain has impacted your ability to make trips away from home, where you would be gone for more than 2-hours?*  *How often does this occur?* | *What did you choose and why?*  *What would be the difference in interference if you selected ‘somewhat’ and ‘quite a bit’* |  |
| **PAININ29:** How often was your pain so severe you could think of nothing else?  Never / Rarely / Sometimes / Often / Always | *Is there anything unclear about the way this question is worded? Please explain.* | *Can you tell me about times where your pain is so severe that you could think of nothing else?*  *How often does this happen?* | *What did you choose and why?*  *What is the difference between rarely and sometimes?* |  |
| **PAININ50:** How often did pain prevent you from sitting for more than 30-minutes?  Never / Rarely / Sometimes / Often / Always | *Is there anything unclear about the way this question is worded? Please explain.* | *Has pain ever impacted your ability to sit for more than 30-minutes? Tell me more about this.*  *Are there any particular reasons why you would find it difficult to sit for more than 30-minutes?* | *What did you choose and why?*  *Do the answers make sense to you?* |  |
| **PAININ47:** How often did pain prevent you from standing for more than 30-minutes?  Never / Rarely / Sometimes / Often / Always | *Is there anything unclear about the way this question is worded? Please explain.* | *Do you experience pain that prevents you from standing for more than 30-minutes? Tell me about that.*  *How often does this happen?*  *Are there any particular reasons why you would find it difficult to stand for more than 30-minutes?* | *What did you choose and why?*  *You chose XXX. What would have to happen for you to choose XXX [one level up or down]* |  |
| **PAININ54:** How often did pain keep you from getting into a standing position?  Never / Once a week or less / Once every few days / Once a day / Every few hours | *Is there anything unclear about the way this question is worded? Please explain.* | *How often did pain affect your ability to get into a standing position over the past 7 days?*  *Has pain ever impacted your ability from getting into a standing position? Tell me about that.*  *Are there any other reasons why you would find it difficult to get into a standing position? Please explain.* | *What did you choose and why?*  *If you experienced this several times in one day only, during a seven day period, what answer would you select?* | *Do you think the timeframe of ‘in the past 7 days’ is appropriate for this question?*  *Was it easy or difficult to remember your experience of pain over the past 7-days?*  *If your pain prevented you from getting into a standing position less frequently at the start of the 7 days, but more frequently in the past 2 days, which response option would you choose and why?* |

- I would now like to ask you some general questions about the Pain Interference questionnaire. While you were answering and discussing those questions I noted down your responses and I am just going to calculate your score now. The answers to each question in the questionnaire is given a score from 1 to 5. To calculate a total score, each of the responses are summed together to get a final score that ranges from 11 to 55 (where higher scores mean worse pain interference). On this questionnaire, your total score was [INSERT PARTICIPANT SCORE]. Please look at the document you were given titled ‘Visual aid for meaningful change questions’ (Appendix E) and look at ‘Figure 3. Pain interference'. This thermometer shows how the scores range for this questionnaire. Would you like to mark that on the thermometer that you received with the questionnaires?
  - - 1. **If you are starting at [participant’s total score], where on the thermometer would you want to get to for you to consider the change important and worth taking a treatment for? Please mark that down.**
  1. *Why would that change be important to you?*
  2. *How would this affect the way you feel?*
  3. *Would this change your day to day life? How?*

1. **Is there anything missing from the questionnaire that you think would be important to [patient term for axSpA] patients?**
2. **Are there any questions which you think should be removed? Maybe questions that don’t apply to you?**
3. **Do you think this questionnaire is good at measuring the pain that you experience because of [patient term for axSpA]? Please explain why.**
4. **How easy or difficult do you find it to answer the questions? Tell me more about that.**
   - *Is there anything that could be done to make this easier?*

## PROMIS Fatigue

- Please take a look at the ‘Fatigue-Short Form’ questionnaire. Please read the instructions out loud. After this, please read each of the questions out loud, tell me your answer and mark down it down on the questionnaire.

(ASK THE PARTICIPANT TO READ EACH INSTRUCTION/QUESTION OUT LOUD BEFORE ASKING THE PROBES PROVIDED FOR EACH INSTRUCTION/QUESTION IN THE TABLE. NOTE DOWN THE PARTICIPANT’S ANSWER FOR EACH QUESTION SO THAT A TOTAL SCORE CAN BE CALCULATED. ALSO ENSURE THE PARTICIPANT IS MARKING DOWN THEIR ANSWERS AS THEY GO ALONG).

| **Instruction/item** | **Understanding** | **Relevance** | **Response options** | **Recall period** |
| --- | --- | --- | --- | --- |
| **INSTRUCTIONS:** Please respond to each question by marking one box per row. | *Is there anything unclear about the way this instruction is worded? Please explain.* |  |  |  |
| **ITEM STEM:** In the past 7 days… |  |  |  |  |
| **HI7**: I feel fatigued  Not at all / A little bit / Somewhat / Quite a bit / Very much | *Is there anything unclear about the way this question is worded? Please explain.* | *Do you ever feel fatigued because of your [patient term for axSpA]?*  *How fatigued do you feel?* | *What did you choose and why?*  *You chose XXX – what would have to happen for you to choose XXX (chose a response option one level up or down)* | *What time-period were you thinking about when answering this question?*  *Do you think the timeframe of ‘in the past 7 days’ is appropriate for this question?*  *If your fatigue was less severe at the start of the 7 days, but more severe in the past 2 days, which response option would you choose and why?* |
| **AN2:** I feel tired  Not at all / A little bit / Somewhat / Quite a bit / Very much | *Is there anything unclear about the way this question is worded? Please explain.* | *To what extent do you ever feel tired because of your [patient term for axSpA]?* | *What did you choose and why?*  *Do the answers make sense to you?* |  |
| **AN3:** I have trouble starting things because I am tired  Not at all / A little bit / Somewhat / Quite a bit / Very much | *Is there anything unclear about the way this question is worded? Please explain.* | *Do you ever have trouble starting things because you are tired as a result of your [patient term for axSpA]?*  *What sort of things do you have trouble starting? (work-related tasks, household work etc.)*  *How much of a problem is this in your life?* | *What did you choose and why?*  *What is the difference between ‘a little bit’’ and ‘somewhat’ trouble starting things because you are tired?* |  |
| **AN4:** I have trouble finishing things because I am tired  Not at all / A little bit / Somewhat / Quite a bit / Very much | *Is there anything unclear about the way this question is worded? Please explain.* | *Do you ever have trouble finishing things because of tiredness as a result of your [patient term for axSpA]?*  *What sort of things do you have trouble finishing? (work-related tasks, household work etc.)*  *How often does this occur?* | *What did you choose and why?* |  |
| **AN5:** I have energy  Not at all / A little bit / Somewhat / Quite a bit / Very much | *Is there anything unclear about the way this question is worded? Please explain.* | *To what extent do you ever feel like you do not have energy because of your [patient term for axSpA]?* | *What did you choose and why?* |  |
| **AN7:** I am able to do my usual activities  Not at all / A little bit / Somewhat / Quite a bit / Very much | *Is there anything unclear about the way this question is worded? Please explain.* | *Are you able to undertake your usual activities?*  *What type of usual activities have been impacted by your axSpA, if ever?*  *To what extent does your [patient term for axSpA] impact your ability to under your usual activities?* | *What did you choose and why?* |  |
| **AN8:** I need to sleep during the day  Not at all / A little bit / Somewhat / Quite a bit / Very much | *Is there anything unclear about the way this question is worded? Please explain.* | *Do you ever need sleep during the day because of your [patient term for axSpA]?*  *How often does this occur?*  *How long do you need to sleep for?* | *What did you choose and why?* |  |
| **AN14:** I need help doing my usual activities  Not at all / A little bit / Somewhat / Quite a bit / Very much | *Is there anything unclear about the way this question is worded? Please explain.* | *To what extent do you ever need help doing your usual activities because of your [patient term for axSpA]?*  *What type of usual activities have you needed help with, if ever?* | *What did you choose and why?*  *What is the difference between ‘a little bit’’ and ‘somewhat’ with you needing help doing your usual activities?* |  |
| **AN15:** I am frustrated by being too tired to do the things I want to do  Not at all / A little bit / Somewhat / Quite a bit / Very much | *Is there anything unclear about the way this question is worded? Please explain.* | *Do you ever get frustrated by being too tired to do the things you want to do?*  *How often does this happen?* | *What did you choose and why?* |  |
| **AN16:** I have to limit my social activity because I am tired  Not at all / A little bit / Somewhat / Quite a bit / Very much | *Is there anything unclear about the way this question is worded? Please explain.* | *To what extent do you to have to limit your social activity because you are tired?*  *What type of social activities have been impacted by being too tired?* | *What did you choose and why?* | *What time-period were you thinking about when answering this question?*  *Was it easy or difficult to remember your experience of fatigue over the past 7-days?*  *Do you think the timeframe of ‘in the past 7 days’ is appropriate for these questions?* |

- I would now like to ask you some general questions about the Fatigue-Short Form questionnaire. While you were answering and discussing those questions I noted down your responses and I am just going to calculate your score now. The answers to each question in the questionnaire is given a score from 1 to 5. To calculate a total score, each of the responses are summed together to get a final score that ranges from 10 to 50 (where higher scores mean worse tiredness and fatigue). On this questionnaire, your total score was [INSERT PARTICIPANT SCORE]. Please look at the document you were given titled ‘Visual aid for meaningful change questions’ (Appendix E) and look at ‘Figure 1. PROMIS fatigue visual aid’. This thermometer shows how the scores range for this questionnaire. Would you like to mark your total score on the thermometer that you received with the questionnaires?

1. **If you are starting at [participant’s total score], where on the thermometer would you want to get to for you to consider the change important and worth taking a treatment for? Please mark that down.**
   1. *Why would that change be important to you?*
   2. *How would this affect the way you feel?*
   3. *Would this change your day to day life? How?*
2. **Is there anything missing from the questionnaire that you think would be important to [patient term for axSpA] patients?**
3. **Are there any questions which you think should be removed? Maybe questions that don’t apply to you?**
4. **Do you think this questionnaire is good at measuring the fatigue/tiredness that you experience because of [patient term for axSpA]? Please explain why.**
5. **How easy or difficult do you find it to answer the questions? Tell me more about that.**
   1. *Is there anything that could be done to make this easier?*

## PROMIS Sleep Disturbance

- Please now take a look at the ‘Sleep Disturbance’ questionnaire. Please read the instructions out loud. After this, please reach each of the questions out loud, tell me your answer and mark down it down on the questionnaire.

(ASK THE PARTICIPANT TO READ EACH INSTRUCTION/QUESTION OUT LOUD BEFORE ASKING THE PROBES PROVIDED FOR EACH INSTRUCTION/QUESTION IN THE TABLE. NOTE DOWN THE PARTICIPANT’S ANSWER FOR EACH QUESTION SO THAT A TOTAL SCORE CAN BE CALCULATED. ALSO ENSURE THE PARTICIPANT IS MARKING DOWN THEIR ANSWERS AS THEY GO ALONG).

| **Instruction/item** | **Understanding** | **Relevance** | **Response options** | **Recall period** |
| --- | --- | --- | --- | --- |
| **INSTRUCTIONS:** Please respond to each question by marking one box per row. | *Is there anything unclear about the way this instruction is worded? Please explain.* |  |  |  |
| **ITEM STEM:** In the past 7 days… |  |  |  |  |
| **Sleep108**: My sleep was restless  Not at all / A little bit / Somewhat / Quite a bit / Very much | *Is there anything unclear about the way this question is worded? Please explain.* | *Do you ever experience restless sleep because of your [patient term for axSpA]?*  *How often do you experience this?* | *What did you choose and why?*  *You chose XXX – what would have to happen for you to choose XXX (chose a response option one level up or down)* |  |
| **Sleep115**: I was satisfied with my sleep  Not at all / A little bit / Somewhat / Quite a bit / Very much | *Is there anything unclear about the way this question is worded? Please explain.* | *Can you tell me about your satisfaction with your sleep?* | *What did you choose and why?*  *Do the answers make sense to you?* | *What time-period were you thinking about when answering this question?*  *Do you think the timeframe of ‘in the past 7 days’ is appropriate for this question?*  *If you were more satisfied with your sleep at the start of the 7 days, but less satisfied in the past 2 days, which response option would you choose and why?* |
| **Sleep44:** I had difficulty falling asleep  Not at all / A little bit / Somewhat / Quite a bit / Very much | *Is there anything unclear about the way this question is worded? Please explain.* | *To what extent do you have difficulties falling asleep because of your [patient term for axSpA]?*  *Is there anything that makes it particularly difficult for you to fall asleep? Please explain.* | *What did you choose and why?*  *What is the difference between ‘a little bit’ and ‘somewhat’ difficulty falling asleep?* |  |
| **Sleep71:** I had trouble getting into a comfortable position to sleep  Not at all / A little bit / Somewhat / Quite a bit / Very much | *Is there anything unclear about the way this question is worded? Please explain.* | *How often do you have trouble getting into a comfortable position to sleep because of your [patient term for axSpA]?* | *What did you choose and why?* |  |
| **Sleep110:** I got enough sleep  Never / Rarely / Sometimes / Often / Always | *Is there anything unclear about the way this question is worded? Please explain.* | *How often do you feel that you do not get enough sleep because of your [patient term for axSpA]?*  *How many days over the past week do you feel you got enough sleep?* | *What did you choose and why?*  *You chose XXX – what would have to happen for you to choose XXX (chose a response option one level up or down)* |  |
| **Sleep92:** I woke up and had trouble falling back to sleep  Never / Rarely / Sometimes / Often / Always | *Is there anything unclear about the way this question is worded? Please explain.* | *Do you ever wake up during the night and then have trouble getting back to sleep because of your [patient term for axSpA]?*  *How long do you typically spend awake after waking during the night because of your axSpA?*  *How often does this happen?* | *What did you choose and why?*  *Do the answers make sense to you?* |  |
| **Sleep109:** My sleep quality was  Very poor / Poor / Fair / Good / Very good | *Is there anything unclear about the way this question is worded? Please explain.* | *Does your [patient term for axSpA] ever affect your sleep quality?*  *How often does this happen?* | *What did you choose and why?*  *What is the difference between ‘poor’ and ‘fair’ sleep quality?* | *Do you think the timeframe of ‘in the past 7 days’ is appropriate for this question?*  *Was it easy or difficult to remember your experience of sleep over the past 7-days?*  *If your sleep quality was good at the start of the 7 days, but became worse in the past 2 days, which response option would you choose and why?* |

- I would now like to ask you some general questions about the Sleep Disturbance questionnaire. While you were answering and discussing those questions I noted down your responses and I am just going to calculate your score now. The answers to each question in the questionnaire is given a score from 1 to 5. To calculate a total score, each of the responses are summed together to get a final score that ranges from 7 to 35 (where higher scores mean worse sleep disturbance). On this questionnaire, your total score was [INSERT PARTICIPANT SCORE]. Please look at the document you were given titled ‘Visual aid for meaningful change questions’ (Appendix E) and look at ‘Figure 2. Sleep disturbance’. This thermometer shows how the scores range for this questionnaire. Would you like to mark your total score on the thermometer that you received with the questionnaires?

1. **If you are starting at [participant’s total score], where on the thermometer would you want to get to for you to consider the change important and worth taking a treatment for? Please mark that down.**
   1. *Why would that change be important to you?*
   2. *How would this affect the way you feel?*
   3. *Would this change your day to day life? How?*
2. **Is there anything missing from the questionnaire that you think would be important to [patient term for axSpA] patients?**
3. **Are there any questions which you think should be removed? Maybe questions that don’t apply to you?**
4. **Do you think this questionnaire is good at measuring the sleep problems that you experience because of [patient term for axSpA]? Please explain why.**
5. **How easy or difficult do you find it to answer the questions? Tell me more about that.**
   1. *Is there anything that could be done to make this easier?*
6. **Do you think all three of these questionnaires are appropriate to ask people with [patient term for axSpA]? Please explain why.**

**End of interview**

*Thank the participant for taking part in the interview and answer any questions they may have.*
